# Crystallographic Engineering Enables Fast Low-Temperature Ion Transport of TiNb_2_O_7_ for Cold-Region Lithium-Ion Batteries

**DOI:** 10.1007/s40820-025-01949-0

**Published:** 2026-01-01

**Authors:** Lihua Wei, Shenglu Geng, Hailu Liu, Liang Deng, Yiyang Mao, Yanbin Ning, Biqiong Wang, Yueping Xiong, Yan Zhang, Shuaifeng Lou

**Affiliations:** 1 State Key Laboratory of Space Power-Sources, School of Chemistry and Chemical Engineering, Harbin Institute of Technology, Harbin, 150001 People’s Republic of China; 2 China Tower Corporation Limited No, 9 Dongran North Street, Haidian District, Beijing, 100089 People’s Republic of China

**Keywords:** Lithium-ion batteries, Low-temperature conditions, Crystallographic engineering, TiNb_2_O_7_, Structure stability

## Abstract

TiNb_2_O_7_ represents an up-and-coming anode material for fast-charging lithium-ion batteries, but its practicalities are severely impeded by slow transfer rates of ionic and electronic especially at the low-temperature conditions. Herein, we introduce crystallographic engineering to enhance structure stability and promote Li^+^ diffusion kinetics of TiNb_2_O_7_ (TNO). The density functional theory computation reveals that Ti^4+^ is replaced by Sb^5+^ and Nb^5+^ in crystal lattices, which can reduce the Li^+^ diffusion impediment and improve electronic conductivity. Synchrotron radiation X-ray 3D nano-computed tomography and in situ X-ray diffraction measurement confirm the introduction of Sb/Nb alleviates volume expansion during lithiation and delithiation processes, contributing to enhancing structure stability. Extended X-ray absorption fine structure spectra results verify that crystallographic engineering also increases short Nb-O bond length in TNO-Sb/Nb. Accordingly, the TNO-Sb/Nb anode delivers an outstanding capacity retention rate of 89.8% at 10 C after 700 cycles and excellent rate performance (140.4 mAh g^−1^ at 20 C). Even at −30 °C, TNO-Sb/Nb anode delivers a capacity of 102.6 mAh g^−1^ with little capacity degeneration for 500 cycles. This work provides guidance for the design of fast-charging batteries at low-temperature condition.

**Supplementary Information:**

The online version contains supplementary material available at 10.1007/s40820-025-01949-0.

## Highlights

- Sb element is introduced into TiNb_2_O_7_ successfully.
- Such crystallographic engineering can narrow the bandgap and broaden the Li^+^ transport channel, making TNO-Sb/Nb electrode possess a better low-temperature performance.
- Such a synergy effect enables TNO-Sb/Nb with large reversible capacity, superior rate performance (140.4 mAh g^−1^ at 20 C), and a high durability of 500 cycles even at −30 °C, holding brand promises in practical applications.

**Supplementary Information:**

The online version contains supplementary material available at 10.1007/s40820-025-01949-0.

## Graphical abstract

**Figure:**
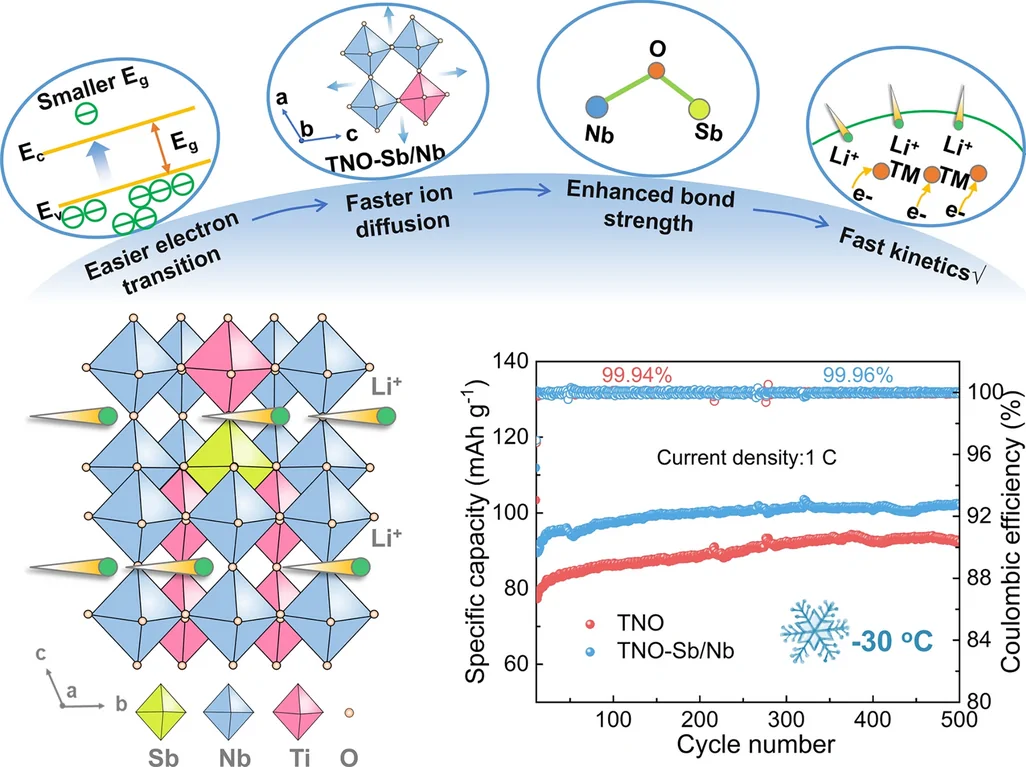

## Introduction

Lithium-ion batteries (LIBs) are regarded as the most encouraging energy storage systems by virtue of exceptional cycle life and high energy density [1, 2]. Anode materials represent a critical challenge in advancing LIBs technology [3]. Graphite, as a result of its excellent specific capacity and low cost, has been extensively used as a commercial anode material in LIBs [4]. Nevertheless, graphite typically has a safety risk of dendrite formation, which is associated with low operating voltages [5, 6]. Especially in fast-charging and low-temperature operation, the danger increases significantly as result of high polarization, which leads to graphite anode insertion potentials below 0 V and causes it hard to charge batteries restricted by cutoff voltage [7–9]. Li_4_Ti_5_O_12_, as a promising anode material, exhibits larger working voltage (1.55 V), rapid Li^+^ transport kinetics, and good low-temperature performance, contributing to cold-region performance and enhanced safety by the suppression of lithium dendrite formation and solid electrolyte interphase (SEI). However, the relatively poor theoretical capacity (175 mAh g^−1^) restricts its commercial applicability [10]. Thus, exploring anode materials with high safety, superior rate performance, excellent low-temperature performance, and a significantly high theoretical capacity for fast-charging LIBs is critical.

TiNb_2_O_7_ (TNO) is first put forward by Goodenough and has been deemed as a promising anode material due to its exceptional structural stability and restrictions on the formation of lithium dendrites [11–13]. Meanwhile, the theoretical specific capacity of TNO is 387.6 mAh g^−1^ due to the high valence states of Ti^4+^ and Nb^5+^, which can undergo five-electron transfer during the electrochemical reaction [13–15]. Though theoretical specific capacity of TNO is comparable to graphite, TNO exhibits unsatisfactory rate capability and cycling lifespan because of its intrinsic low ionic and electronic conductivities. Nanosized or nanoarchitectured design is an effective method to enhance electrochemical reactions by reducing ion diffusion lengths [16–19], whereas TNO possesses the lower compaction density and volume capacity on account of more lattice voids and porous morphology, coupled with the close working potential to the decomposition window of the electrolyte which aggravates the interfacial side reactions, posing significant challenges for practical adoption [20, 21]. Doping engineering represents a straightforward approach to enhance ion and electron transport kinetics in TiNb_2_O_7_-based anodes [20, 22–26], exemplified by compounds such as 5Cu-TNO@NC [27], Tb_0.01_-TNO [28], Ce_0.01_-TNO [15, 29], Zr_0.05_-TNO [30], and Fe_5_-TNO [17]. Nevertheless, there are relatively few studies on the effect of crystallographic engineering on low-temperature property of TNO, which is important for expanding its application potential in the fields of low-temperature energy storage, solid electrolyte, or superconducting materials. Therefore, we employ Sb/Nb crystallographic engineering to boost the electronic/ionic conductivity of TNO and simultaneously investigate its effect on the low-temperature performance of TNO. Several reasons highlight the choice of Sb and Nb. Above all, the raw material of the dopant Sb_2_O_3_ is a non-toxic and low-cost commercial material. Secondly, the Sb element can not only reduce the bandgap to increase electronic conductivity, but also participates in redox reaction of Sb^5+^/Sb^3+^ to realize double electron transfer per Sb, which contributes to the improvement in theoretical and practical capacity. In addition, there will be an increase in ionic conductivity, considering self-doping Nb^5+^ (0.64 Å) at Ti^4+^ (0.605 Å) sites is beneficial to expand the ion transport channel.

Herein, Sb/Nb crystallographic engineering is introduced in the bulk of Wadsley-Roth phase TNO to effectively enhance electronic conductivity and accelerate Li^+^ transport kinetics. In situ X-ray diffraction (XRD) and density functional theory (DFT) calculation reveals the structural changes and the kinetic laws of Li⁺ storage and transfer. Meanwhile, the effect of Sb/Nb crystallographic engineering on the low-temperature performance of TNO is investigated. The incorporation of Sb^5+^ enhances the valence bond strength and the increased Nb^5+^ broadens Li^+^ diffusion channels, which remarkably improve the structure stability, ion diffusion, and charge mobility. Benefitting from that, the capacity retention of the TNO-Sb/Nb electrode is 89.8% after 700 cycles at 10 C and low-temperature ability (−30 °C) with almost no capacity decay after 500 cycles at 1 C. Furthermore, the constructed TNO-Sb/Nb||NCM pouch cell exhibits excellent rechargeable performance (1.14 Ah at 17 C), confirming that this crystallographic design effectively broadens the application of TNO anode. This study provides a solution to the problem of poor low-temperature performance of TNO and offers new ideas for the development of next-generation fast-charging long-life LIBs.

## Experiments and Method

### Synthesis of Anode Materials

The TiNb_2_O_7_ (TNO) and Ti_0.97_Sb_0.015_Nb_2.015_O_7_ (TNO-Sb/Nb) materials are prepared by a simple solid-state reaction. Mix TiO_2_ (99.9%), Nb_2_O_5_ (99.9%), and Sb_2_O_3_ (99.99%) in a certain stoichiometric number, then calcine at 1100 °C for 24 h, and then, obtain the TNO and TNO-Sb/Nb materials after cooling to room temperature.

### Materials Characterization

The X-ray diffraction (XRD) with Cu kα radiation is collected in the Bruker D8 Advance diffractometer range from 10° to 80°. The morphology and microstructure of TNO and TNO-Sb/Nb are visualized by scanning electron microscope (SEM, HELIOS Nanolab 600i). High-resolution transmission electron microscope (HRTEM) images and X-ray energy-dispersive spectrum (EDS) maps are examined by the transmission electron microscope (TEM, FEI Talos 200S). X-ray photoelectron spectroscopy (XPS, Thermo Scientific K-Alpha) is mainly applied for quantitative analysis of elemental chemical states. The inductively coupled plasma (ICP) tests are implemented to quantitatively analyze the composition of materials. The X-ray microtomography experiments are operated at the nano-3D imaging line station (BL18BB). The energy at this work is 20 keV, and the distance between the sample and the detector is 10 cm. Image data of the samples are collected with an angle range of 0° ~ 180°. The complete tomographic scan dataset is gathered for about 60 min with 900 projections (each projection exposure time is 3 s). The resolution of the CT images is 650 nm/pixel. After the original data are aligned by tomography, the 3D electrode is reconstructed, and data visualization and analysis are performed in the software package Avizo. Nb K-edge X-ray absorption measurements are conducted at the rare element analysis line station (BL13SSW), and the obtained data are analyzed by using the software package Athena software, Artemis, and Hephaestus.

### Electrochemical Measurements

To evaluate electrochemical performance of TNO and TNO-Sb/Nb composites, they are used as active materials of anodes for assembling CR2032 coin-type half-cells, which are examined on the Neware-CT3008 system. Electrodes consist of polyvinylidene fluoride (PVDF) binder, Super P, and active composites with mass ratio of 1:1:8 and mix in *N*-methyl-2-pyrrolidone; subsequently, the uniformly mixed slurry is coated on Cu foil and then dried at 80 °C for 10 h in the vacuum. Anode pieces are procured from the foil cut into disks with an areal mass loading of active materials of 1.4 ~ 1.9 mg cm^−2^. The Li half-cells are assembled in an argon-filled glove box (H_2_O < 0.01 ppm; O_2_ < 0.01 ppm). The separator is Celgard 2500 polypropylene membrane, and the liquid electrolyte is LiPF_6_ (1 M) in a mixture of EC/DEC/DMC (1:1:1 in volume). Galvanostatic discharge-charge and GITT are tested by using Neware-CT3008 system in a range of 1.0-3.0 V. The CHI760 electrochemical workstation is used to implement EIS and CV tests.

### Preparation of Pouch Cell

The active material of the cathode is LiNi_0.8_Co_0.1_Mn_0.1_O_2_ (NCM), and both electrodes are made up of 10 wt% PVDF binder, 80 wt% active materials, as well as 10 wt% Super P. For the TNO-Sb/Nb||NCM pouch cell, the loading mass of cathode and anode electrodes is 16.0 and 20.7 mg cm^−2^, respectively. The amount of electrolyte is 14 mL. The 1 C-rate is determined as 1.7 Ah at the voltage range of 1.5 ~ 2.75 V. The energy densities of TNO-Sb/Nb||NCM pouch cell are calculated by the equations: volumetric energy density = cell capacity × average voltage/cell volume, and gravimetric energy density = cell capacity × average voltage/cell weight. To avoid the formation of lithium dendrites caused by insufficient anode materials to receive the Li^+^ transferred from the cathode materials, excessive anode materials are adopted to ensure the safety of cell. However, too much anode materials will reduce the energy density of cell, so the N/P ratio of the TNO-Sb/Nb||NCM pouch cell is set to 1.05/1.

### Calculation Section

The DFT calculations are employed on electronic structure analysis, energetic calculation, and structural optimization by using projector-augmented wave (PAW) method in the Vienna ab initio simulation package (VASP). The spin polarization generalized gradient approximation (GGA) of Perdew-Burke-Ernzerhof (PBE) can be acquired from electron-ion exchange-correlation functional. Brillouin-zone integrations are sampled by employing k-point mesh of a Gamma-centered 1 × 3 × 2. The electronic ion interaction is defined by the PAW pseudopotentials. The valence electron states are amplified in the plane wave basis set, with a cutoff energy of 500 eV. The electron energy is self-consistent when the energy change is lower than 10^–6^ eV, while the force changes lower than 0.03 eV Å^−1^ are used to determine the convergence of geometric optimization. Li^+^ ion migration is calculated by using a 1 × 3 × 1 TiNb_2_O_7_ supercell bulk model. The adsorption/desorption of EC-Li is evaluated by using the TiNb_2_O_7_ (001) slab model with a *p*(1 × 3) supercell. After structural optimization, it is found that the doped Sb and Nb mainly occupy the Ti sites.

## Results and Discussion

### Construction and Characterization

**Fig. 1:**
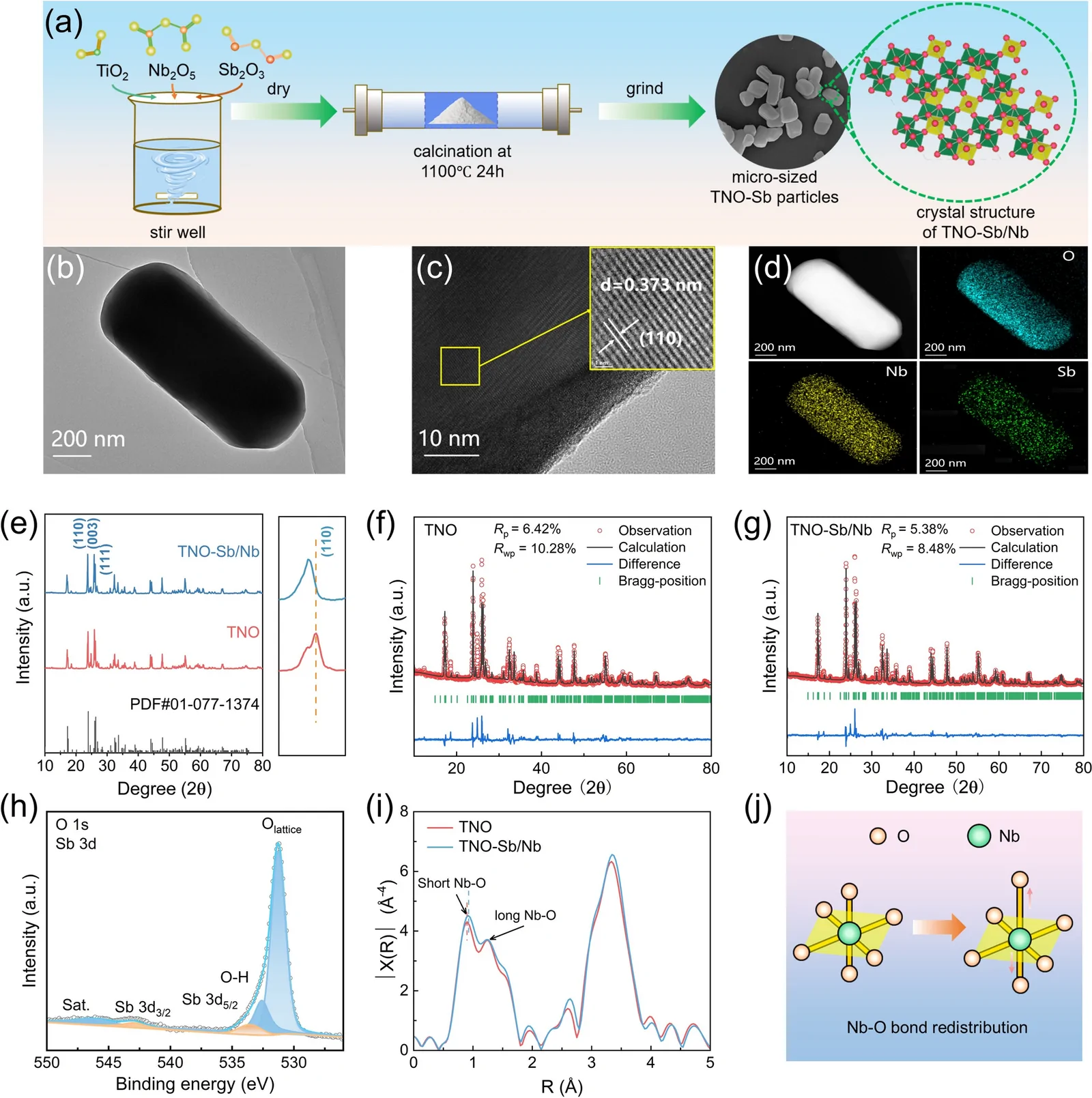
Preparation, microstructural analysis, and compositional characterization of the TNO and TNO-Sb/Nb. **a** Schematic synthesis of the preparation for TNO-Sb/Nb. **b** TEM image of the TNO-Sb/Nb microrods. **c** HRTEM image of the TNO-Sb/Nb. **d** STEM image and the corresponding EDS mappings of TNO-Sb/Nb. **e** XRD patterns and partial enlarged views for TNO and TNO-Sb/Nb. The Rietveld refinement of XRD patterns of **f** TNO and **g** TNO-Sb/Nb. **h** XPS high-resolution spectra of TNO-Sb/Nb for O 1*s* and Sb 3*d*. **i** EXAFS spectra of the TNO and TNO-Sb/Nb. **j** Diagram of octahedral changes induced by Nb-O bond redistribution

TNO and TNO-Sb/Nb are prepared by a simple high-temperature solid phase method (Fig. 1a**)**. As shown in Fig. S1, TNO-Sb/Nb and TNO consist of typical rod-like morphology measuring 500 nm ~ 2 μm. The microstructures of TNO and TNO-Sb/Nb are elucidated by the TEM (Figs. 1b and **S2a**) and HRTEM (Figs. 1c and **S2b**), which suggest the interplanar crystal spacings of TNO and TNO-Sb/Nb are 0.365 and 0.373 nm, respectively, both matched well with the (110) crystal planes. Compared with TNO, TNO-Sb/Nb shows larger interplanar spacings, indicating the increased unit cell volume which is more conducive to the diffusion of Li^+^. Selected area electron diffraction (SAED) patterns (**Fig. S2c, d**) verify fine single-phase structure and crystallinity of TNO-Sb/Nb and TNO. In addition, the elemental mapping consequences prove that the contained elements are evenly distributed in rod-like particles of TNO and TNO-Sb/Nb, and Sb element has been successfully incorporated into TNO-Sb/Nb (Figs. 1d, **S3**, and **S4**). The ICP results show the content of each element in the TNO and TNO-Sb/Nb materials and further confirm the co-doping of Sb and Nb (Table S1).

To explore the crystal structure of TNO and TNO-Sb/Nb, XRD measurements are employed. The XRD patterns of the two materials (Fig. 1e) can be matched well with monoclinic phase ReO_3_ (JCPDS-77-1374) without observing any impurities phase. It is noteworthy that as the doping amount of Sb and Nb enlarges from 0 to 0.015, all the diffraction peaks transfer to a lower angle (**Fig. S5**), signifying an augment on interplanar spacing by crystallographic engineering, which is well consistent with the aforementioned HRTEM results. To further understand the lattice structure change of TNO and TNO-Sb/Nb samples, the Rietveld refinement is performed (Fig. 1f, g). The results concluded that the TNO-Sb/Nb (*a* = 20.4192 Å and *c* = 11.9192 Å) shows larger lattice parameters (**Table S2**), implying that TNO-Sb/Nb can provide wider Li^+^ diffusion channels compared to TNO, which facilitates faster diffusion and storage of Li^+^.

To analyze the elemental composition, electronic structure, and chemical state of material surfaces, XPS is presented (Figs. 1h and **S6**). The peaks around 464.2 and 458.4 eV belong to Ti 2*p*_1/2_ and Ti 2*p*_3/2_ of Ti^4+^, respectively. Meanwhile, we can see that the introduction of Sb/Nb causes the Ti spectra slightly move to smaller binding energies, which may be induced by the reduction of atoms through charge redistribution. The detected peaks located at 533.6 and 542.9 eV (Fig. 1h) match well with Sb 3*d*_5/2_ and Sb 3*d*_3/2_ of Sb^5+^, respectively, further confirming the existence of Sb in the lattice of TNO-Sb/Nb. To objectively prove the existence of the redox activity of Sb, the XPS high-resolution spectra are conducted at different states of charge (SOC). As manifested in **Figs. S7** and** S8**, when discharging to 2 V, the XPS spectra of Sb 3*d* and Nb 3*d* for TNO-Sb/Nb show a clear energy transfer. For instance, the binding energy of Sb 3*d*_5/2_ peak for TNO-Sb/Nb transfers from 533.6 to 533.2 eV, along with the binding energy of Sb 3*d*_3/2_ peak moves from 542.9 to 542.5 eV, revealing the reduced valence state of Sb and part of Sb^5+^ components are transformed into Sb^3+^. When discharging to 1.5 V and 1 V, due to the dense SEI films form on the surface as discharging time prolongs, there are no peaks of Sb/Nb elements are detected. Interestingly, when charging back to 3 V (**Fig. S7c**), the peaks of Sb 3*d*_5/2_ and Sb 3*d*_3/2_ are restored to 533.6 and 542.9 eV, respectively, which suggests that Sb^3+^ participates in the oxidation reaction. These results validate that Sb participates in the redox process and provides Sb^5+^/Sb^3+^ electron pair during cycling. The X-ray absorption analysis is conducted to verify that Sb/Nb crystallographic engineering changes the distribution of niobium ions (**Fig. S9a**). The Nb K-edge X-ray absorption near edge spectroscopy (XANES) spectra slightly move to higher energy (**Fig. S9b**), certifying the increased valence state of Nb for TNO-Sb/Nb. The extended X-ray absorption fine structure (EXAFS) spectra of Nb reveal the coexistence of long Nb-O and short Nb-O bonds in two samples (Fig. 1i). After Sb/Nb crystallographic engineering, peak area and intensity of short Nb-O peak at the lowest R-space [15] slightly increase and shift, manifesting the short Nb-O bond is getting longer after redistribution (Fig. 1j).

### Electronic Structure and Li^+^ Diffusion Mechanism

**Fig. 2:**
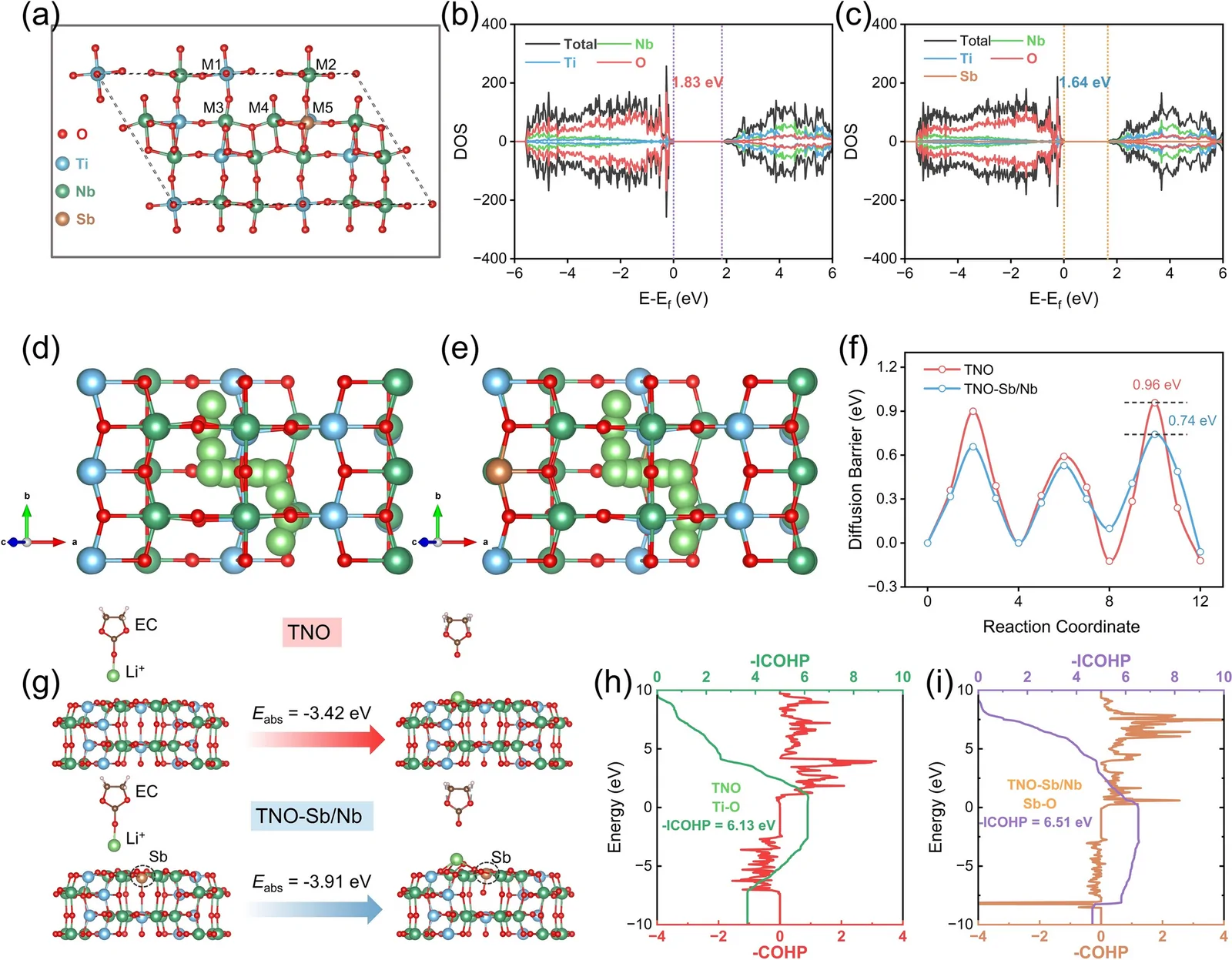
**a** Optimized structure model of TNO-Sb/Nb for DOS. Density of states calculation results for **b** TNO and **c** TNO-Sb/Nb. The Li^+^ diffusion models of **d** TNO and **e** TNO-Sb/Nb. **f** Diffusion barrier of Li^+^ along b tunnel of TNO and TNO-Sb/Nb. **g** Interfacial Li^+^ adsorption energy of TNO and TNO-Sb/Nb. Crystal orbital Hamilton population curves (COHP) for **h** Ti-O and **i** Sb-O bonds

To acquire in-depth understanding of the effect of Sb/Nb crystallographic engineering on the intrinsic electronic structure and the mechanism of Li^+^ diffusion, DFT computations are performed. The substitution sites of Sb and Nb are initially judged based on earlier articles [14, 31, 32] and ultimately determined by the thermodynamically lowest energy occupied sites. As highlighted in Fig. 2a, the Ti occupied site in TNO can be classified into five types; structural optimization results show that the introducing Sb and Nb elements tend to occupy the M5 site, where the structure exhibits lowest energy and is most stable. Generally, narrow bandgaps are conduced to the formation of intrinsic electrons and holes. As illustrated in Fig. 2b, c**,** the bandgap value of TNO-Sb/Nb is 1.64 eV, which is minor than that of TNO (1.83 eV). This can be ascribed to the introduce of Sb shift the energy most closely to Fermi level of conduction band to the left [28], which narrows the bandgap, improving the conductivity and electrochemical reaction kinetics. Except for the research of electronic conductivity for TNO-Sb/Nb, the Li^+^ diffusion barriers and the diffusion channels for TNO-Sb/Nb and TNO are also evaluated in Fig. 2d–f. Notably, the energy barrier of Li^+^ diffusion along *b*-axis for TNO-Sb/Nb is reduced compared to TNO (Fig. 2f). With the Sb/Nb crystallographic engineering, the Li^+^ diffusion barrier reduces dramatically from 0.96 to 0.74 eV. These calculation results can be attributed to the increase in lattice parameters *a* and *c*, which prompts the broadened Li^+^ diffusion channels along the *b* axis, consequently promoting the Li^+^ migration during electrochemical reactions.

Furthermore, the corresponding adsorption energies of TNO and TNO-Sb/Nb are calculated to analyze the effect of Sb/Nb crystallographic engineering on the charge storage capacity of electrode materials (Fig. 2g). The adsorption energy (*E*_ads_) of the TNO-Sb/Nb surface with solvated Li-EC (−3.91 eV) is significantly larger than the unmodified TNO surface (−3.42 eV), indicating the Sb/Nb crystallographic engineering can improve surface adsorption capacity by optimizing the electronic and geometric structure, thereby facilitating pseudocapacitive charge storage. The Ti-O and Sb-O bonding states are studied by crystal orbital Hamilton population (COHP) computation. In general, a positive –COHP value represents a bonding state and a negative –COHP value corresponds to an antibonding state, respectively [33]. To quantitatively evaluate the bond strength changes induced by Sb/Nb crystallographic engineering, the negative integrated COHP (–ICOHP) is implemented. The high –ICOHP value represents the strong orbital hybridization of the bond. As displayed in Fig. 2h, i, the –ICOHP value of Sb-O bond (6.51 eV) is larger than Ti-O (6.13 eV), demonstrating the stronger bond strength of TNO-Sb/Nb, concurrently identifying its superior structural stability.

### Electrochemical Performances

**Fig. 3:**
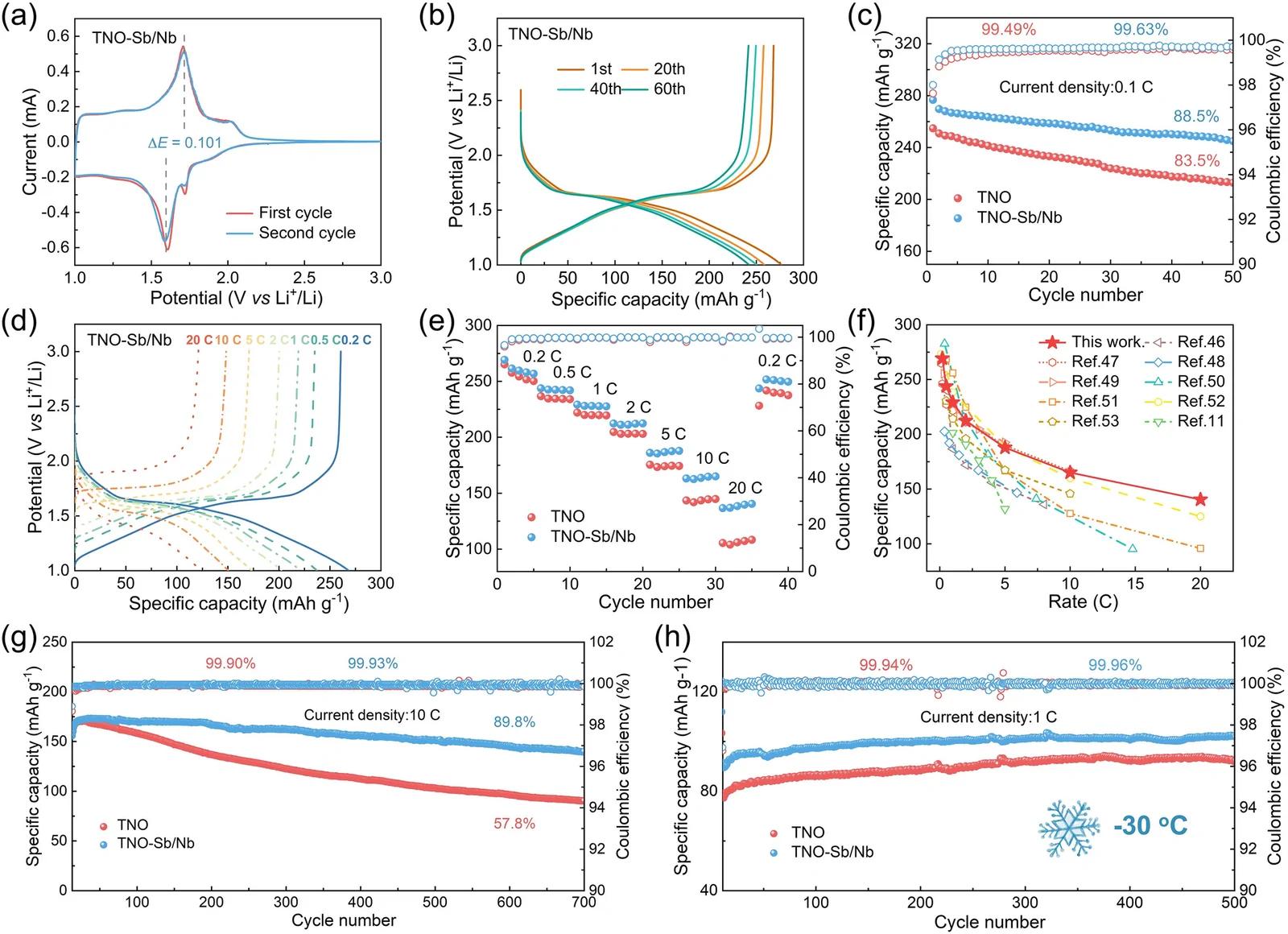
Electrochemical properties of TNO-Sb/Nb and TNO in half cell configuration: **a** CV curves of TNO-Sb/Nb within 1-3 V during the initial two turns. **b** Charge-discharge curves for TNO-Sb/Nb cells with different number of cycles at 0.1 C in 1.0 ~ 3.0 V. **c** Cycling performance during 50 cycles at 0.1 C of TNO and TNO-Sb/Nb. **d** Charge-discharge curves of TNO-Sb/Nb at different current densities. **e** Rate performance. **f** Performance comparison of TNO-Sb/Nb with TNO-based composites previously reported [11, 46–53]. **g** Cycling performance during 700 cycles at 10 C of TNO-Sb/Nb and TNO electrodes. **h** Cycling performance of TNO and TNO-Sb/Nb at 1 C in −30 °C

The electrochemical performances of TNO and TNO-Sb/Nb are researched by cyclic voltammetry (CV). As displayed in Figs. 3a and **S10**, three obvious couples of anodic and cathodic peaks of TNO-Sb/Nb and TNO are observed in the CV curves. The peaks at 1.60 V and 1.70 V demonstrate the presence of Nb^5+^/Nb^4+^ redox reactions. The peaks centered at 1.90/2.03 and 1.25/1.32 V can be identified with the redox reactions of Ti^4+^/Ti^3+^ and Nb^4+^/Nb^3+^, respectively. The almost coincident CV curves manifest the highly reversible kinetics of the two electrodes. These reversible redox couples are closely correlated with the intercalation/deintercalation behaviors of Li^+^, confirming a certain structural stability and relatively balanced reaction dynamics of electrode materials. Remarkably, the redox potential difference in the first cycle of TNO-Sb/Nb (101 mV) is smaller than TNO (125 mV) (**Fig. S10**), revealing the smaller polarization and better reversibility during Li^+^ intercalation/deintercalation, accounting for the superior rate and cycling performance of TNO-Sb/Nb (as will be demonstrated later). Figures 3b and **S11a** illustrate the evolutions of the charge-discharge profiles for both anodes over the 60 cycles at 0.1 C in 1 ~ 3 V. It can be obtained that TNO and TNO-Sb/Nb exhibit a pair of conspicuous and highly reversible voltage plateaus during the cycle, which represents the phase transition. But the voltage plateaus of TNO decay significantly faster compared to TNO-Sb/Nb, revealing the higher structural stability of TNO-Sb/Nb. Meanwhile, the voltage curves of TNO-Sb/Nb remain relatively stable with minimal changes and show the lower capacity fading, while the voltage curves of TNO display more significant attenuation. This is mainly rooted in the smaller charge transfer impedance and diffusion resistance of TNO-Sb/Nb, coupled with the reduction in interface side reactions.

The cycle performances of TNO and TNO-Sb/Nb are investigated at 0.1 and 1 C (1 C = 270 mA g^−1^) as depicted in Figs. 3c and **S12**. Compared with TNO (254.8 mAh g^−1^, 83.5%), TNO-Sb/Nb exhibits the higher specific capacity (276.7 mAh g^−1^) and capacity retention (88.5%) at 0.1 C after 50 cycles. Meanwhile, the premier coulombic efficiency of TNO-Sb/Nb is about 98.0%, slightly larger than that of TNO (97.6%). Interestingly, the average coulombic efficiency of TNO-Sb/Nb is higher than that of TNO for long-circulation at any current density. This represents the more reversible extraction of lithium from the crystalline structure for TNO-Sb/Nb. Moreover, TNO-Sb/Nb electrode is characterized by an outstanding first reversible specific capacity of 244.2 mAh g^−1^, corresponding to 1.05 Li^+^/TM is reversibly intercalated at 1 C, as well as reveals a capacity retention of 87.6% after 300 cycles. As far as TNO, the capacity is 0.94 Li^+^/TM (219.7 mAh g^−1^) and the capacity retention is 87.4% over 300 cycles at 1 C. Furthermore, the TNO-Sb/Nb also displays a better rate performance (Figs. 3d, e and **S11b**). Capacity retention rate of TNO-Sb/Nb is about 52.2% when the current density aggrandizes from 0.2 to 20 C, which is higher than TNO (40.8%), implying the higher capacity and power density of TNO-Sb/Nb. The discharge capacities of TNO-Sb/Nb sample deliver 269.2, 243.8, 229.1, 212.3, 187.8, 165.0, and 140.4 mAh g^−1^ at 0.2, 0.5, 1, 2, 5, 10, and 20 C, respectively. There is a capacity retention rate up to 93.5% (251.7 mAh g^−1^) for TNO-Sb/Nb when the current density comes back to 0.2 C. On the contrary, the specific capacity of TNO decreases to 108.3 mAh g^−1^ at 20 C, and the capacity retention rate is only 90.3% (239.4 mAh g^−1^) when the current density comes back to 0.2 C. The rate performance of the present TNO-Sb/Nb anode compares favorably with those of the reported TNO optimized anodes for LIBs (Fig. 3f). On the whole, the superior rate performance is promoted by excellent conductivity and reduced Li^+^ diffusion barrier in TNO-Sb/Nb, which can be demonstrated by the previous calculation results.

The cycling performances of TNO-Sb/Nb and TNO are also researched at high current density, as highlighted in Fig. 3g, where a respectable cycling stability for TNO-Sb/Nb can be achieved with 89.8% capacity retention at the end of 700 cycles at 10 C. Even at a lower temperature of −30 °C, TNO-Sb/Nb can still exhibit a high capacity of 102.6 mAh g^−1^ at 1 C, which is superior to TNO (92.3 mAh g^−1^). Benefitting from the facilitated Li^+^ diffusion kinetics and accelerated electron transfer, TNO-Sb/Nb has almost no decay in specific capacity and an excellent average coulombic efficiency of 99.96% for 500 cycles (Fig. 3h). As depicted in **Fig. S13**, TNO-Sb/Nb anode achieves a specific capacity of 123.3 mAh g^−1^ after 500 cycles at 1 C in −20 °C with no capacity degradation. While TNO anode only exhibits a specific capacity of 109.3 mAh g^−1^ with a capacity retention rate of 88.8% after 263 cycles at 1 C in −20 °C. When the temperature drops to −40 °C, TNO-Sb/Nb anode still delivers a specific capacity of 101.0 mAh g^−1^ and a capacity retention of 100% after 150 cycles at 0.2 C, which is surpassing than TNO (85.6 mAh g^−1^, 100%). Such results validate that the Sb/Nb crystallographic engineering dramatically improves the low-temperature performance of TNO materials. At high temperatures, as affected by the high-temperature decomposition of electrolyte, the specific capacity of TNO and TNO-Sb/Nb decays faster (**Fig. S14**). Consequently, we will further conduct high-temperature modification researches on TNO-Sb/Nb in future to enhance its operational durability at high temperatures.

### Electrochemical Reaction Kinetics

To analyze the difference of surface chemistry between pristine TNO and doped TNO-Sb/Nb samples after cycling, XPS depth profiling analysis is carried out by using argon ion etching to characterize the atomic concentration of elements after 300 cycles at 1 C (**Fig. S15**). Notably, there are no Nb 3*d* signal peaks detected on the surface of TNO, while TNO-Sb/Nb still displays obvious signal peaks of Nb 3*d* spectra. It is not difficult to infer that both TNO and TNO-Sb/Nb can form the dense SEI films during discharging process (**Figs. S8a-d and S15**). However, compared to TNO-Sb/Nb, TNO holds poorer degradability on SEI films during charging, causing TNO the thicker SEI films after multiple cycles; thus, no peaks of Nb 3*d* spectra are recognized. As the etching depth increases, there are not only Nb^5+^ but also un-oxidized Nb^3+^ discovered in the TNO and TNO-Sb/Nb, and the content of un-oxidized Nb^3+^ is growing. The un-oxidized Nb^3+^ illustrates the presence of dead lithium in electrode materials. The thicker the SEI films form, the more the dead lithium is generated, throwing light upon the reason of more severe capacity decay for TNO electrode after cycling.

**Fig. 4:**
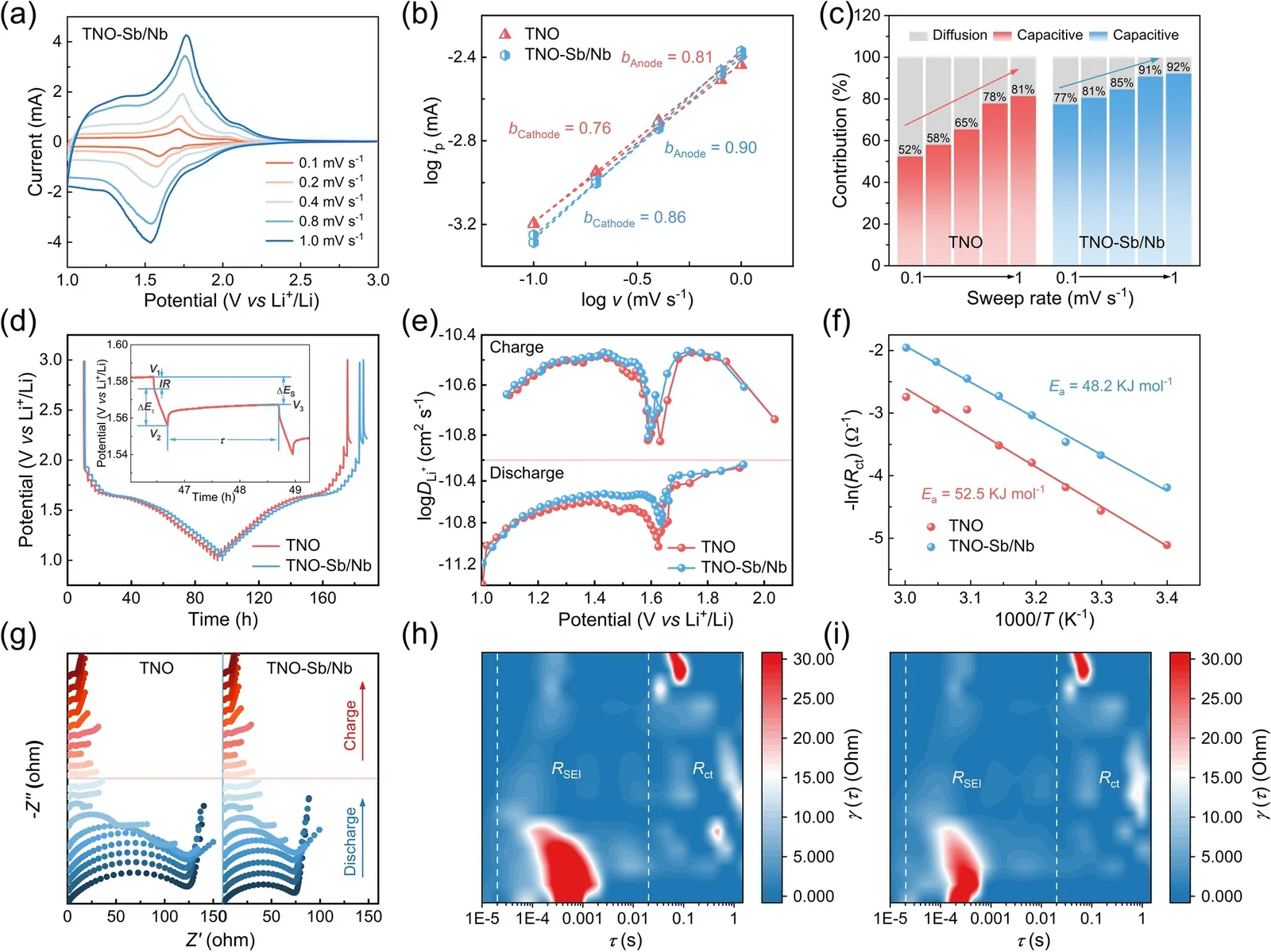
Kinetics analysis: **a** CV curves of the TNO-Sb/Nb under various scan rates range from 0.1 to 1.0 mV s^−1^. **b** Relation diagrams of the log *i*_p_ and log *v* for TNO and TNO-Sb/Nb electrodes. **c** Proportional distribution of pseudo-capacitance contribution and diffusion contribution capacities at various sweep rates. **d** GITT curves. **e** Computed results of Li^+^ diffusion coefficients for TNO and TNO-Sb/Nb. **f** Arrhenius plots and calculated activation energy. **g** EIS evolution at different potentials. The corresponding two-dimensional DRT diagram for **h** TNO and **i** TNO-Sb/Nb

To investigate the reaction kinetics of the TNO and TNO-Sb/Nb, CV tests are performed. As displayed in Figs. 4a and **S16a**, the shapes of the CV curves are consistent at different scanning rates, indicating that the TNO-Sb/Nb electrode has excellent stability. It is not difficult to find that there is a linear relationship between the square root of scanning rate (*ν*^1/2^) and the peak current (*i*_p_) (**Fig. S16b, c**). The slope absolute values of the *i*_p_-*ν*^1/2^ fitting lines for TNO-Sb/Nb are larger, revealing it has a greater Li^+^ diffusion coefficient as a result of the expanded lattice volume induced by the Sb/Nb crystallographic engineering. The pseudo-capacitance contribution of the TNO-Sb/Nb is calculated from the equation relating current and sweep rate [34]:

1 $$i = av^{b}$$

where both *a* and *b* are variables. Particularly, the reaction dynamic is dominated by diffusion control behavior when the calculated *b* value approaches 0.5. In contrast, reaction dynamic is dominated by pseudocapacitive electrochemistry when the *b* value draws near 1.0. The* b* value can be obtained by the slope of the log*i*_p_-log*v* diagram, as depicted in Fig. 4b. The *b* values in the anodic and cathodic processes for TNO and TNO-Sb/Nb cells are 0.81/0.76 and 0.90/0.86, respectively, suggesting a predominance of surface or near-surface controlled fast charge storage over diffusion-controlled bulk phase processes. The contributions of the above two control modes are further quantified by Eq. (2) [35, 36]:

2 $$i = k_{1} v + k_{2} v^{\frac{1}{2}}$$

where *k*_2_*v*^1/2^ denotes the diffusion contribution and *k*_1_*v* denotes the capacitive contribution. The pseudo-capacitance process is a reversible and rapid redox process which occurs at or near the surface without phase transition or chemical bond breaking. The capacitive contribution of TNO-Sb/Nb at 0.2 mV s^−1^ reaches as high as 81% (**Fig. S17b**), which is much more than that of TNO (58%) (**Fig. S17a**). Further tests show the capacitive contribution of TNO-Sb/Nb is higher than TNO at diverse sweep speeds (Fig. 4c). What’s more, the capacitive contribution of TNO-Sb/Nb is even up to 92% at 1 mV s^−1^. The higher pseudocapacitive contribution means faster electrode reaction kinetics of TNO-Sb/Nb, which unveils the fundamental reason for the favorable rate performance of the TNO-Sb/Nb electrodes.

To elucidate the Li^+^ diffusion kinetics for TNO and TNO-Sb/Nb electrodes, galvanostatic intermittent titration technique (GITT) is implemented. Fluctuations of *D*_Li_^+^ values during the whole electrochemical process stem from the extraction and insertion of Li^+^ from different sites [37]. As summarized in Fig. 4d, e, the calculated *D*_Li_^+^ values stay in the magnitude range of 10^−10^ ~ 10^−12^ cm^2^ s^−1^. It should be noted that the *D*_Li_^+^ values of TNO-Sb/Nb are higher than that of TNO, manifesting that the TNO-Sb/Nb electrode is blessed with the accelerated Li^+^ diffusion during charge and discharge processes. To more in-depth research Li^+^ diffusion kinetics for TNO and TNO-Sb/Nb electrodes at low temperatures, the GITT tests at −30 °C are conducted. As depicted in **Fig. S18**, TNO-Sb/Nb still carries a greater Li^+^ diffusion coefficient at low temperatures by virtue of the decreased diffusion energy barriers and widened Li^+^ diffusion channels induced by Sb/Nb crystallographic engineering, resulting in the reduced polarization. In consequence, the Sb/Nb crystallographic engineering remarkably improves the low-temperature performance of TNO-Sb/Nb.

The charge transfer kinetics of TNO-Sb/Nb are further explored by experimental method. As we all know, activation energy of charge transfer can be obtained by the Arrhenius equation [38]:

3 $$\frac{1}{{R_{{{\mathrm{ct}}}} }} = {\mathrm{A}}_{0}^{{ - \frac{{E_{{\mathrm{a}}} }}{RT}}}$$

in which *R*_ct_ is the charge transfer impedance, *R* belongs to gas constant,* T* represents the temperature (K), A_0_ defines as a constant number, and *E*_a_ constitutes the activation energy. The *R*_ct_ is assessed by the electrochemical impedance spectroscopy (EIS) tests at various temperatures (**Fig. S19**). As illustrated in **Fig. S20**, the equivalent circuit is identified based on the EIS pattern, where *R*_Ω_, *Z*_w_, and *R*_ct_ belong to electrolyte resistance, Warburg impedance, and charge transfer impedance, respectively. *R*_ct_ results of TNO and TNO-Sb/Nb fitted by the equivalent circuit are shown in **Table S3**. As the *R*_ct_ of TNO-Sb/Nb at different temperatures is smaller than TNO, we can conclude that TNO-Sb/Nb possesses faster interfacial reaction and lower energy barrier [39]. According to the Arrhenius equation, the calculated activation energy (*E*_a_) of TNO-Sb/Nb is 48.2 kJ mol^−1^, which is lower than TNO (52.5 kJ mol^−1^), indicating the proportion of activated molecules has increased, thereby promoting Li^+^ reaction kinetics (Fig. 4f).

To more deeply comprehend the Li^+^ kinetic evolution process than the EIS plots (Fig. 4g), the distribution of relaxation times (DRT) method is applied [40, 41]. The DRT is a technique that is able to transform the EIS in the frequency domain into the DRT function in the time domain by Eq. (4) [42]:

4 $$Z\left( \omega \right) = R_{\infty } + \mathop \smallint \limits_{0}^{\infty } \frac{\gamma \left( \tau \right)}{{1 + j\omega \tau }}d\tau$$

Peak area indicates impedance value, and a peak of particular relaxation time represents a related specialized electrochemical process. As highlighted in **Fig. S21**, the DRT results can distinctly distribute the electrochemical phase transitions of the TNO and TNO-Sb/Nb electrodes and their several evolutions on impedance at different SOC. The peaks at 2 × 10^−5^ to 2 × 10^−2^ s show an irreversible evolution that is derived from the SEI formation and ion transport at the interface SEI [43]. It is worth noting that the *R*_SEI_ values in the charging process are more stable and smaller compared with the discharging process, which is related to the degradation of the SEI films during charging process. Moreover, the *R*_SEI_ values of TNO-Sb/Nb are smaller than TNO, accounting for the larger average coulombic efficiency of TNO-Sb/Nb cycling at different current densities. Relaxation processes at 2 × 10^−2^ to 20 s are relatively reversible, representing the charge transfer processes (*R*_ct_) [44]. The TNO-Sb/Nb electrode has lower *R*_ct_, coinciding well with the lower activation energies for charge transfer (Fig. 4h, i). DRT mappings reveal that TNO-Sb/Nb and TNO own similar phase transition processes and impedance evolution at different SOC; nevertheless, TNO-Sb/Nb exhibits smaller impedance values and earlier electrode reaction (Figs. 4h, i** and S22**), which indicate the smaller polarization and faster electrode reaction kinetics for TNO-Sb/Nb after introducing the Sb/Nb crystallographic engineering. Even at −30 °C, where impedance values of both materials increase owing to the decreased electronic conductivity and enlarged viscosity of electrolyte, TNO-Sb/Nb still expresses lower* R*_SEI_ and *R*_ct_ values than those of TNO. This can be ascribed to the Sb/Nb crystallographic engineering which lowers the activation energy, narrows the band gap, and expands the Li^+^ transport channels, thereby reducing polarization and improving the ion/electron transport for TNO-Sb/Nb at low temperatures (**Fig. S22**). Together, these results explain the superior rate performance and low-temperature performance observed for the TNO-Sb/Nb electrode.

### Electrode Structure Evolution and Stability Analysis

**Fig. 5:**
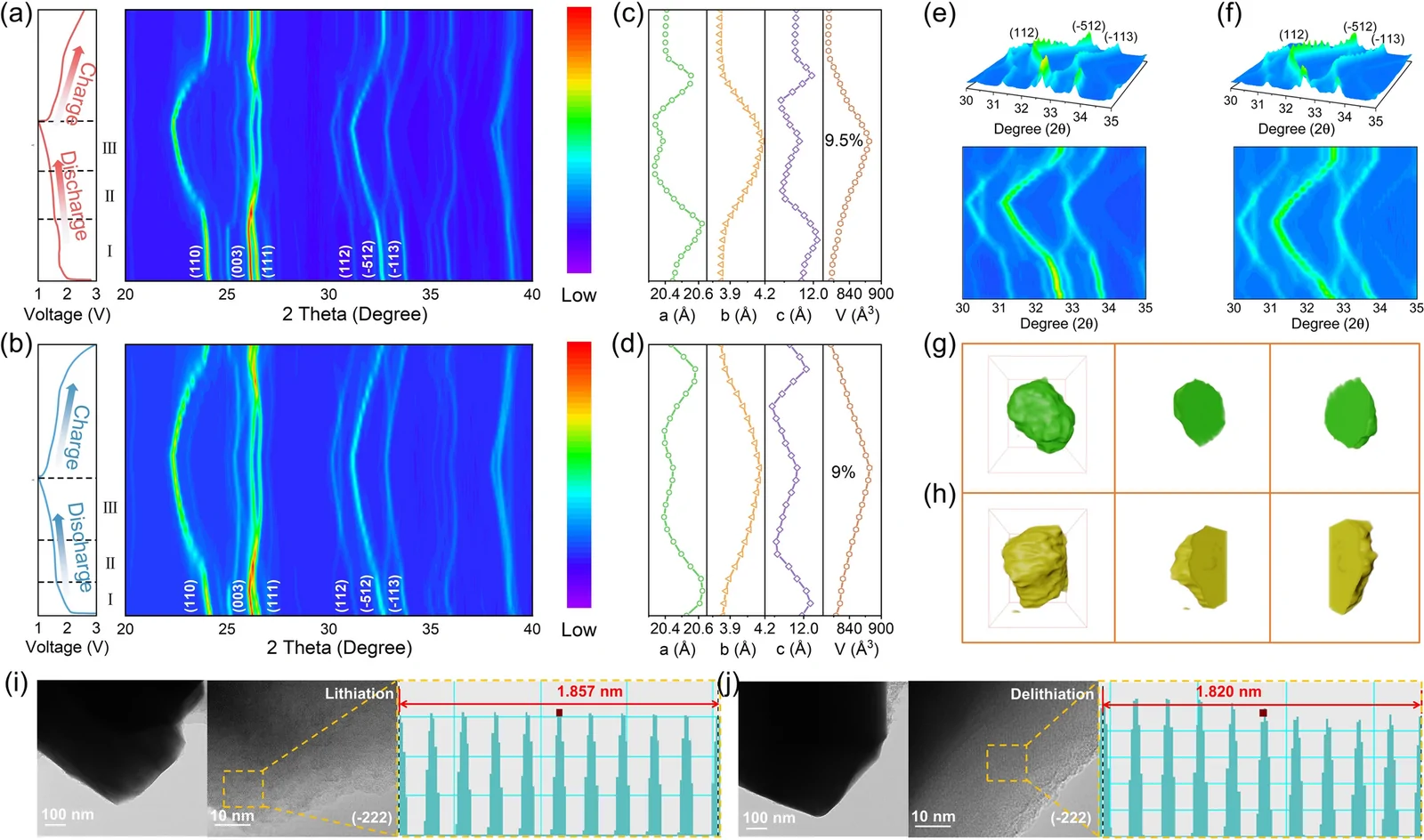
2D contour maps of in situ XRD patterns of **a** TNO and **b** TNO-Sb/Nb for the first cycle at 67.5 mA g^−1^. Lattice constant variations in** c** TNO and **d** TNO-Sb/Nb. 3D surface maps and corresponding projection drawing for **e** TNO and **f** TNO-Sb/Nb. The 3D images of TNO-Sb/Nb **g** before and **h** after cycling by synchrotron radiation X-ray 3D nano-computed tomography. TEM and HRTEM images for TNO-Sb/Nb microspheres at **i** discharge to 1.0 V and **j** charge to 3.0 V along (−222) zone axis

In situ XRD analysis is implemented to monitor crystal structural evolutions of TNO and TNO-Sb/Nb upon lithiation/delithiation, unraveling significantly different Li-storage behaviors. The structural evolution in the lithiation process includes three regions (Fig. 5a, b). Region I and region III are solid solution phase transition region, in which the voltage curve changes quickly. Region II belongs to the two-phase coexistence zone, where a stable platform appears. As demonstrated in **Fig. S23**, TNO-Sb/Nb and TNO first go through the I region during the discharging process, in which the diffraction peaks of (110), (−111), (401), (003), (111), (112), (−512), (−113), (601), and (511) constantly move toward smaller angles. It is apparent that no new phase diffraction peaks are generated despite the interplanar spacing enlarges, revealing I region is the solid solution phase transition region. After discharging to II region, the diffraction peaks of (110), (−111), (112), (−512), (−113), (511) still deviate to small angles, resulting in the growth of lattice constant *b*. Surprisingly, the intensity of all diffraction peaks in this region gradually weakens, along with an expansion of the half-peak width, signifying that lattice distortion aggrandizes with the constant insertion of Li^+^, which leads to an increase in the degree of lattice disorder. Meanwhile, some characteristic diffraction peaks such as (003), (111), (110), (−512), (401), (601) fade and form the new peaks by cause of the redistribution of atoms and changes of chemical composition. Therefore, the II region is two-phase coexistence zone. At end of the II region, all the characteristic peaks disappear and convert to new phase (Li_1.68_TNO or Li_1.68_TNO-Sb/Nb) [19]. When discharging to III region, the vast majority of new characteristic peaks migrate to small angles and gently intensify with the insertion of Li^+^, while no other characteristic peaks are observed, verifying that the III region is a solid solution phase transition region. As a result, the lattice constants *a*, *b*, and *c* in this region are constantly increasing as the volume of the unit cell increases (Fig. 5c, d). The phase evolution pathway of charging process is opposite. It can be clearly observed that all the diffraction peaks return to their original positions along the opposite phase transition path when the charging is completed, approving the excellent reversibility of the structure, which explains the reason for their good long-cycle performance.

The evolution of the lattice parameters is extracted by Rietveld refinements, as presented in Fig. 5c, d. By the Sb/Nb crystallographic engineering, TNO-Sb/Nb presents a lower change of lattice parameters than TNO in the lithiation and delithiation processes. According to the 2D contour plots and 3D surface colormaps, TNO-Sb/Nb shows lower offset than those of TNO (Fig. 5e, f). In situ XRD refinement displays that the cell volume expansion coefficients of TNO-Sb/Nb at 1.0 V is 9%, lower than 9.5% for TNO. Note that the *b* value increases monotonically throughout discharge process, which means that the Li^+^ diffusion path is along the *b*-axis perpendicular to the *ac*-plane. The more gently lattice parameter change and smaller volume expansion are owing to the broaden of ion diffusion channels and the stronger bond strength after introducing the Sb/Nb crystallographic engineering, which is conducive to the enhancement of long-term cycling ability.

To further clarify superior long-term mechanical stability of TNO-Sb/Nb, the nanoindentation test is carried out. As shown in **Tables S4** and **S5**, the average elastic modulus and average hardness of the TNO-Sb/Nb materials after 300 cycles at 1 C are 9.5 and 0.13 GPa, respectively, which are larger than those of the TNO (5.4 and 0.07 GPa). The results establish that TNO-Sb/Nb possesses good mechanical strength, which can resist stress impact and plastic deformation during cycling, thus enhancing the long-term structural stability. To study the effect of Sb/Nb crystallographic engineering on the stress-strain in the bulk phase of TNO-Sb/Nb materials after circulation, synchrotron radiation X-ray 3D nano-computed tomography is conducted. It is important to note that no cracks are observed before and after cycling, hinting that TNO-Sb/Nb owns a super-stable structural framework, which is capable of withstanding the volumetric strain during long-term Li^+^ intercalation/de-intercalations (Fig. 5g, h) [45]. To further verify that the TNO-Sb/Nb possesses small stress and strain after circulation, the TEM and HRTEM are investigated. As shown in Fig. 5i, j, TEM and HRTEM images of the TNO-Sb/Nb reveal that lithiation and delithiation along (−222) zone axis demonstrates an inconsiderable variation in the interplanar spacing, manifesting an excellent small-strain characteristic and the reversible electrochemical redox reaction of TNO-Sb/Nb, which further confirms the superior structural stability during Li^+^ insertion/extraction process. This is responsible for the better cycling stability of the TNO-Sb/Nb anode.

To explore the long-term thermodynamic stability of TNO-Sb/Nb anode under repeated cycling and temperature fluctuations, the surface morphology, microstructure and change of component for the TNO-Sb/Nb are elucidated after 500 cycles at 1 C in −30 °C. To be noted, there are no fractures appearing on the TNO-Sb/Nb electrode (**Fig. S24**). Simultaneously, the post-cycling TEM, post-cycling STEM, and post-cycling elemental mapping results denote that TNO-Sb/Nb materials keep intact, crack-free particle morphology with uniform elemental distribution after low-temperature cycling (**Fig. S25**). Additionally, TNO-Sb/Nb electrode shows no impurity phases formation, hinting there is no dopant migration or phase segregation occurring over extended use (**Fig. S26**). Such observations convince that TNO-Sb/Nb owns a splendid structural robustness even after prolonged low-temperatures cycling.

### Practical Application

**Fig. 6:**
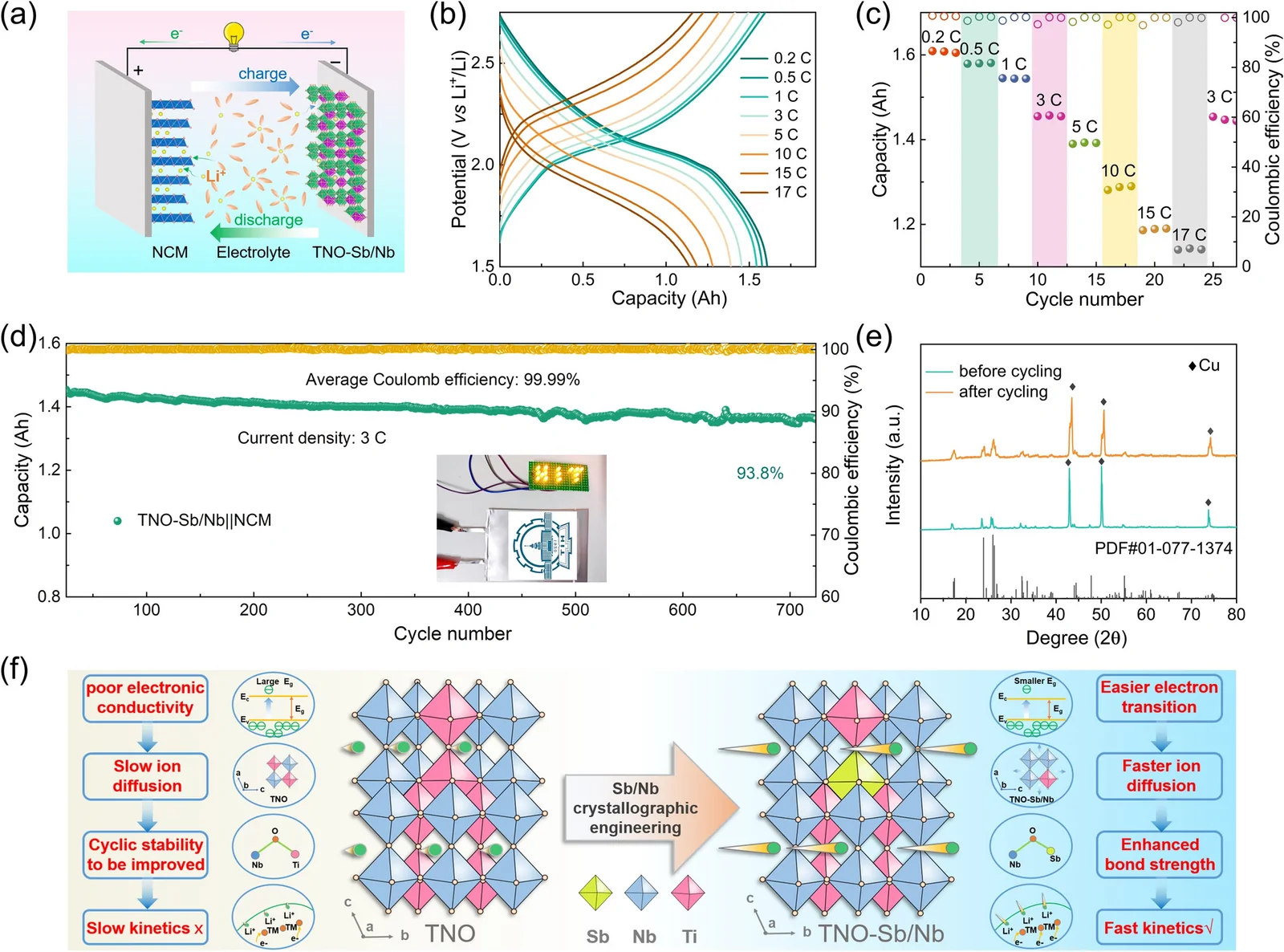
Electrochemical properties of TNO-Sb/Nb||NCM pouch cell: **a** Schematical illustration of TNO-Sb/Nb||NCM pouch cell. **b** Charge/discharge curves at varying rates. **c** Rate performance. **d** Cycle stability of TNO-Sb/Nb||NCM at 3 C (The inset shows the pouch cell lighting a LED). **e** XRD patterns before and after the cycle for TNO-Sb/Nb||NCM pouch cell. **f** Schematic illustration revealed the different lithiation kinetics mechanisms before and after the crystallographic engineering

Aiming at further exploring the practical applicability of TNO-Sb/Nb, a pouch cell of TNO-Sb/Nb||LiNi_0.8_Co_0.1_Mn_0.1_O_2_ (NCM) is assembled (Fig. 6a). Figure 6b and c presents the excellent rate performance of TNO-Sb/Nb||NCM pouch cell with average capacities of 1.61, 1.58, 1.54, 1.46, 1.39, 1.29, 1.19, and 1.14 Ah at 0.2, 0.5, 1, 3, 5, 10, 15, and 17 C, respectively. It is noteworthy that the average specific capacity of 1.45 Ah is regained when the current density is reverted to 3 C, which is 99.3% of the previous capacity, verifying its wonderful reversibility. Besides, the pouch cell shows good cycling stability even at 3 C, which maintains an impressive capacity retention ratio of 93.8% after 700 cycles, with a capacity loss of only 0.0089% per cycle (Fig. 6d). Moreover, TNO-Sb/Nb||NCM pouch cell acquires a gravimetric energy density of 94.4 Wh kg^−1^ at 3 C and a volumetric energy density of 243.1 Wh L^−1^ at 0.2 C. The results of XRD patterns reveal that there is no impurity phase generation before and after the cycle, proving that no side reaction occurs during the cycle (Fig. 6e). In light of the above experimental and theoretical analyses, we explore and rationalize these intertwined reaction processes and their determining factors, and simultaneously draw the different lithiation kinetics mechanisms diagram to provide inspiration for the structural design of the next generation of long-life and fast-charging batteries (Fig. 6f).

## Conclusion

In summary, a comprehensive study has been performed on the Sb and Nb bimetallic synergistic optimized TiNb_2_O_7_-based anode materials to understand the influence of the co-introduction of Sb/Nb on the electronic structure and the lattice structure. DFT calculation demonstrates that the Sb/Nb crystallographic engineering improves the electrical conductivity and decreases the Li^+^ diffusion barrier of TiNb_2_O_7_. In situ XRD and synchrotron radiation X-ray 3D nano-computed tomography certify the crystallographic engineering reduces the volume and strain change of the unit cell upon repeated cycling, facilitating the structural stability. Therefore, TNO-Sb/Nb electrode exhibits prominent cycling stability and high-rate performance, with a capacity loss of only 0.015% per cycle over 700 cycles at 10 C and a capacity of 140.4 mAh g^−1^ at 20 C. At −30 °C, TNO-Sb/Nb electrode presents preferable low-temperature performance with negligible capacity decline after 500 cycles at 1 C. Moreover, the TNO-Sb/Nb||NCM pouch cell still achieves a remarkable capacity retention ratio of 93.8% after 700 cycles at 3 C. Our work employing crystallographic engineering design provides a new approach to develop high-performance electrode materials for high-power and low-temperature LIBs.

## Supplementary Information

Figures of TEM and SEM images, HRTEM image, SEAD patterns, STEM image and EDS mappings, XPS high-resolution spectra, XAS spectra and XANES spectra, CV curves, charge-discharge curves, cycling performances, cyclic voltammograms, EIS results, linear fitting results of* i*_p_ vs.* v*^1/2^, pseudocapacitive contributions, equivalent circuit, and DRT profiles. Table of Rietveld refinement results and equivalent circuit fitting data. Below is the link to the electronic supplementary material.

Supplementary file1 (DOC 29726 KB)

## Declarations

### Supporting Information

Figures of TEM and SEM images, HRTEM image, SEAD patterns, STEM image and EDS mappings, XPS high-resolution spectra, XAS spectra and XANES spectra, CV curves, charge-discharge curves, cycling performances, cyclic voltammograms, EIS results, linear fitting results of *i*_p_ vs. *v*^1/2^, pseudocapacitive contributions, equivalent circuit, and DRT profiles. Table of Rietveld refinement results and equivalent circuit fitting data.

## References

[1] C.-Y. Wang, T. Liu, X.-G. Yang, S. Ge, N.V. Stanley et al., Fast charging of energy-dense lithium-ion batteries. Nature **611**(7936), 485–490 (2022). 10.1038/s41586-022-05281-0 36224388 10.1038/s41586-022-05281-0

[2] J. Liu, M. Yue, S. Wang, Y. Zhao, J. Zhang, A review of performance attenuation and mitigation strategies of lithium-ion batteries. Adv. Funct. Mater. **32**(8), 2107769 (2022). 10.1002/adfm.202107769

[3] M. Weiss, R. Ruess, J. Kasnatscheew, Y. Levartovsky, N.R. Levy et al., Fast charging of lithium-ion batteries: a review of materials aspects. Adv. Energy Mater. **11**(33), 2101126 (2021). 10.1002/aenm.202101126

[4] L. Zhao, B. Ding, X.-Y. Qin, Z. Wang, W. Lv et al., Revisiting the roles of natural graphite in ongoing lithium-ion batteries. Adv. Mater. **34**(18), 2106704 (2022). 10.1002/adma.202106704 10.1002/adma.202106704 35032965

[5] J. Bi, Z. Du, J. Sun, Y. Liu, K. Wang et al., On the road to the frontiers of lithium-ion batteries: a review and outlook of graphene anodes. Adv. Mater. **35**(16), 2210734 (2023). 10.1002/adma.202210734 10.1002/adma.202210734 36623267

[6] T. Gao, Y. Han, D. Fraggedakis, S. Das, T. Zhou et al., Interplay of lithium intercalation and plating on a single graphite particle. Joule **5**(2), 393–414 (2021). 10.1016/j.joule.2020.12.020

[7] J. Hou, M. Yang, D. Wang, J. Zhang, Fundamentals and challenges of lithium ion batteries at temperatures between −40 and 60 °C. Adv. Energy Mater. **10**(18), 1904152 (2020). 10.1002/aenm.201904152

[8] Y. Liu, Y. Zhu, Y. Cui, Challenges and opportunities towards fast-charging battery materials. Nat. Energy **4**(7), 540–550 (2019). 10.1038/s41560-019-0405-3

[9] C.-Y. Wang, G. Zhang, S. Ge, T. Xu, Y. Ji et al., Lithium-ion battery structure that self-heats at low temperatures. Nature **529**(7587), 515–518 (2016). 10.1038/nature16502 26789253 10.1038/nature16502

[10] L. Zhang, C. Zhu, S. Yu, D. Ge, H. Zhou, Status and challenges facing representative anode materials for rechargeable lithium batteries. J. Energy Chem. **66**, 260–294 (2022). 10.1016/j.jechem.2021.08.001

[11] J.-T. Han, Y.-H. Huang, J.B. Goodenough, New anode framework for rechargeable lithium batteries. Chem. Mater. **23**(8), 2027–2029 (2011). 10.1021/cm200441h

[12] Y. Yang, J. Zhao, Wadsley-Roth crystallographic shear structure niobium‐based oxides: promising anode materials for high‐safety lithium‐ion batteries. Adv. Sci. **8**(12), 2004855 (2021). 10.1002/advs.202004855 10.1002/advs.202004855 PMC8224428 34165894

[13] Y. Zhang, W. Zhao, C. Kang, S. Geng, J. Zhu et al., Phase-junction engineering triggered built-in electric field for fast-charging batteries operated at −30 °C. Matter **6**(6), 1928–1944 (2023). 10.1016/j.matt.2023.03.026

[14] S. Geng, Y. Zhang, L. Shi, A. Shi, L. Zhou et al., Enabling 20 min fast-charging Ah-level pouch cell by tailoring the electronic structure and ion diffusion in TiNb_2_O_7_. Energy Storage Mater. **68**, 103339 (2024). 10.1016/j.ensm.2024.103339

[15] A. Shi, Y. Zhang, S. Geng, X. Song, G. Yin et al., Highly oxidized state dopant induced Nb-O bond distortion of TiNb_2_O_7_ for extremely fast-charging batteries. Nano Energy **123**, 109349 (2024). 10.1016/j.nanoen.2024.109349

[16] L. Yin, D. Pham-Cong, I. Jeon, J.-P. Kim, J. Cho et al., Electrochemical performance of vertically grown WS_2_ layers on TiNb_2_O_7_ nanostructures for lithium-ion battery anodes. Chem. Eng. J. **382**, 122800 (2020). 10.1016/j.cej.2019.122800

[17] F. Yu, B. Miglani, S. Yuan, R. Yekani, K.H. Bevan et al., Fe^3+^-substitutional doping of nanostructured single-crystal TiNb_2_O_7_ for long-stable cycling of ultra-fast charging anodes. Nano Energy **133**, 110494 (2025). 10.1016/j.nanoen.2024.110494

[18] I. Jeon, L. Yin, D. Yang, H. Chen, S.W. Go et al., Enhanced Li storage of pure crystalline-C_60_ and TiNb_2_O_7_-nanostructure composite for Li-ion battery anodes. J. Energy Chem. **97**, 478–485 (2024). 10.1016/j.jechem.2024.06.004

[19] B. Guo, X. Yu, X.-G. Sun, M. Chi, Z.-A. Qiao et al., A long-life lithium-ion battery with a highly porous TiNb_2_O_7_ anode for large-scale electrical energy storage. Energy Environ. Sci. **7**(7), 2220–2226 (2014). 10.1039/C4EE00508B

[20] H. Wang, R. Qian, Y. Cheng, H.-H. Wu, X. Wu et al., Micro/nanostructured TiNb_2_O_7_-related electrode materials for high-performance electrochemical energy storage: recent advances and future prospects. J. Mater. Chem. A **8**(36), 18425–18463 (2020). 10.1039/D0TA04209A

[21] R. Zhan, S. Liu, W. Wang, Z. Chen, S. Tu et al., Micrometer-scale single crystalline particles of niobium titanium oxide enabling an Ah-level pouch cell with superior fast-charging capability. Mater. Horiz. **10**(11), 5246–5255 (2023). 10.1039/D3MH01160G 37740481 10.1039/d3mh01160g

[22] R. Wang, L. Wang, R. Liu, X. Li, Y. Wu et al., “Fast-charging” anode materials for lithium-ion batteries from perspective of ion diffusion in crystal structure. ACS Nano **18**(4), 2611–2648 (2024). 10.1021/acsnano.3c08712 38221745 10.1021/acsnano.3c08712

[23] L. Hu, L. Luo, L. Tang, C. Lin, R. Li et al., Ti_2_Nb_2__*x*_O_4+5__*x*_ anode materials for lithium-ion batteries: a comprehensive review. J. Mater. Chem. A **6**(21), 9799–9815 (2018). 10.1039/c8ta00895g

[24] H. Song, Y.-T. Kim, A Mo-doped TiNb_2_O_7_ anode for lithium-ion batteries with high rate capability due to charge redistribution. Chem. Commun. **51**(48), 9849–9852 (2015). 10.1039/C5CC02221E 10.1039/c5cc02221e 25989881

[25] Y. Sheng, X. Yue, W. Hao, Y. Dong, Y. Liu et al., Balancing the ion/electron transport of graphite anodes by a La-doped TiNb_2_O_7_ functional coating for fast-charging Li-ion batteries. Nano Lett. **24**(12), 3694–3701 (2024). 10.1021/acs.nanolett.3c05151 38411584 10.1021/acs.nanolett.3c05151 PMC10979427

[26] S. Zhu, M. Su, S. Lu, S. Yang, Y. Huang et al., Enhanced performance of Mo-doped TiNb_2_O_7_ anode material for lithium-ion batteries *via* KOH sub-molten salt synthesis. Appl. Surf. Sci. **669**, 160507 (2024). 10.1016/j.apsusc.2024.160507

[27] C. Yang, D. Ma, J. Yang, M. Manawan, T. Zhao et al., Crystallographic insight of reduced lattice volume expansion in mesoporous Cu^2+^-doped TiNb_2_O_7_ microspheres during Li^+^ insertion. Adv. Funct. Mater. **33**(15), 2212854 (2023). 10.1002/adfm.202212854

[28] Y. Zhang, C. Kang, W. Zhao, B. Sun, X. Xiao et al., Crystallographic engineering to reduce diffusion barrier for enhanced intercalation pseudocapacitance of TiNb_2_O_7_ in fast-charging batteries. Energy Storage Mater. **47**, 178–186 (2022). 10.1016/j.ensm.2022.01.061

[29] G. Yu, J. Huang, X. Bai, T. Li, S. Song et al., Engineering of cerium modified TiNb_2_O_7_ nanoparticles for low-temperature lithium-ion battery. Small **20**(34), e2308858 (2024). 10.1002/smll.202308858 38618927 10.1002/smll.202308858

[30] X. Jin, Y. Deng, H. Tian, M. Zhou, W. Tang et al., Homovalent doping: an efficient strategy of the enhanced TiNb_2_O_7_ anode for lithium-ion batteries. Green Energy Environ. **9**(8), 1257–1266 (2024). 10.1016/j.gee.2023.01.007

[31] C. Yang, C. Lin, S. Lin, Y. Chen, J. Li, Cu_0.02_Ti_0.94_Nb_2.04_O_7_: an advanced anode material for lithium-ion batteries of electric vehicles. J. Power. Sources **328**, 336–344 (2016). 10.1016/j.jpowsour.2016.08.027

[32] J. Gao, X. Cheng, S. Lou, Y. Ma, P. Zuo et al., Self-doping Ti_1-*x*_Nb_2+*x*_O_7_ anode material for lithium-ion battery and its electrochemical performance. J. Alloys Compd. **728**, 534–540 (2017). 10.1016/j.jallcom.2017.09.045

[33] X. Cao, H. Li, Y. Qiao, P. He, Y. Qian et al., Reversible anionic redox chemistry in layered Li_4/7_[□_1/7_Mn_6/7_]O_2_ enabled by stable Li-O-vacancy configuration. Joule **6**(6), 1290–1303 (2022). 10.1016/j.joule.2022.05.006

[34] J. Ma, Y. Xiang, J. Xu, W. Zhang, H. Zhang et al., Reducing lithium-diffusion barrier on the wadsley-Roth crystallographic shear plane *via* low-valent cation doping for ultrahigh power lithium-ion batteries. Adv. Energy Mater. **15**(12), 2403623 (2025). 10.1002/aenm.202403623

[35] T. Brezesinski, J. Wang, S.H. Tolbert, B. Dunn, Ordered mesoporous alpha-MoO_3_ with iso-oriented nanocrystalline walls for thin-film pseudocapacitors. Nat. Mater. **9**(2), 146–151 (2010). 10.1038/nmat2612 20062048 10.1038/nmat2612

[36] S. Lou, X. Cheng, Y. Zhao, A. Lushington, J. Gao et al., Superior performance of ordered macroporous TiNb_2_O_7_ anodes for lithium ion batteries: understanding from the structural and pseudocapacitive insights on achieving high rate capability. Nano Energy **34**, 15–25 (2017). 10.1016/j.nanoen.2017.01.058

[37] L. Zhu, S. Xiang, M. Wang, D. Sun, X. Zhang et al., Lattice regulation boosts working voltage and energy density of Na_3.12_Fe_2.44_(P_2_O_7_)_2_ cathode for sodium-ion batteries. Adv. Funct. Mater. **35**(26), 2419611 (2025). 10.1002/adfm.202419611

[38] B. Peng, Y. Chen, F. Wang, Z. Sun, L. Zhao et al., Unusual site-selective doping in layered cathode strengthens electrostatic cohesion of alkali-metal layer for practicable sodium-ion full cell. Adv. Mater. **34**(6), e2103210 (2022). 10.1002/adma.202103210 34811831 10.1002/adma.202103210

[39] L. Du, Y. Zhang, Y. Xiao, D. Yuan, M. Yao et al., A defect-rich carbon induced built-in interfacial electric field accelerating ion-conduction towards superior-stable solid-state batteries. Energy Environ. Sci. **18**(6), 2949–2961 (2025). 10.1039/D4EE05966B

[40] L. Xu, M. Yao, L. Du, Y. Chen, Y. Wei et al., Accelerating lithium ion conduction *via* activated interfacial dipole layer for long-life and high-voltage solid-state lithium-metal battery. J. Energy Chem. **108**, 92–100 (2025). 10.1016/j.jechem.2025.03.073

[41] Q. Wang, Y. Zhang, M. Yao, K. Li, L. Xu et al., A lithium-selective “OR-gate” enables fast-kinetics and ultra-stable Li-rich cathodes for polymer-based solid-state batteries. Energy Environ. Sci. **18**(6), 2931–2939 (2025). 10.1039/D4EE05264A

[42] Y. Zhang, Y. Chen, M. Yan, F. Chen, Reconstruction of relaxation time distribution from linear electrochemical impedance spectroscopy. J. Power. Sources **283**, 464–477 (2015). 10.1016/j.jpowsour.2015.02.107

[43] Y. Lu, C.-Z. Zhao, R. Zhang, H. Yuan, L.-P. Hou et al., The carrier transition from Li atoms to Li vacancies in solid-state lithium alloy anodes. Sci. Adv. **7**(38), eabi5520 (2021). 10.1126/sciadv.abi5520 34524850 10.1126/sciadv.abi5520 PMC8443184

[44] Y. Zhang, M. Yao, P. Jing, Y. Fu, W. Wang et al., Achieving a highly reversible four-electron redox of S/Cu_2_S for aqueous Zn/S─Cu battery. Angew. Chem. Int. Ed. **137**(25), e202501205 (2025). 10.1002/ange.202501205 10.1002/anie.202501205 40222955

[45] P. Xue, C. Guo, L. Tan, Hydrogen-bonding crosslinking MXene to highly mechanically stable and super-zincophilic host for stable Zn metal anode. Chem. Eng. J. **472**, 145056 (2023). 10.1016/j.cej.2023.145056

[46] S. Li, X. Cao, C.N. Schmidt, Q. Xu, E. Uchaker et al., TiNb_2_O_7_/graphene composites as high-rate anode materials for lithium/sodium ion batteries. J. Mater. Chem. A **4**(11), 4242–4251 (2016). 10.1039/C5TA10510B

[47] A.G. Ashish, P. Arunkumar, B. Babu, P. Manikandan, S. Sarang et al., TiNb_2_O_7_/Graphene hybrid material as high performance anode for lithium-ion batteries. Electrochim. Acta **176**, 285–292 (2015). 10.1016/j.electacta.2015.06.122

[48] Y. Zhang, M. Zhang, Y. Liu, H. Zhu, L. Wang et al., Oxygen vacancy regulated TiNb_2_O_7_ compound with enhanced electrochemical performance used as anode material in Li-ion batteries. Electrochim. Acta **330**, 135299 (2020). 10.1016/j.electacta.2019.135299

[49] G. Wang, Z. Wen, L. Du, Y.-E. Yang, S. Li et al., Hierarchical Ti-Nb oxide microspheres with synergic multiphase structure as ultra-long-life anode materials for lithium-ion batteries. J. Power. Sources **367**, 106–115 (2017). 10.1016/j.jpowsour.2017.09.061

[50] X. Wang, G. Shen, Intercalation pseudo-capacitive TiNb_2_O_7_@carbon electrode for high-performance lithium ion hybrid electrochemical supercapacitors with ultrahigh energy density. Nano Energy **15**, 104–115 (2015). 10.1016/j.nanoen.2015.04.011

[51] H. Li, L. Shen, G. Pang, S. Fang, H. Luo et al., TiNb_2_O_7_ nanoparticles assembled into hierarchical microspheres as high-rate capability and long-cycle-life anode materials for lithium ion batteries. Nanoscale **7**(2), 619–624 (2015). 10.1039/C4NR04847D 25423342 10.1039/c4nr04847d

[52] S. Gong, Y. Wang, Q. Zhu, M. Li, Y. Wen et al., High-rate lithium storage performance of TiNb_2_O_7_ anode due to single-crystal structure coupling with Cr^3+^-doping. J. Power. Sources **564**, 232672 (2023). 10.1016/j.jpowsour.2023.232672

[53] H. Bian, J. Gu, Z. Song, H. Gong, Z. Zhang et al., An effective strategy to achieve high-power electrode by tin doping: Sn_*x*_-TiNb_2_O_7_ as a promising anode material with a large capacity and high-rate performance for lithium-ion batteries. J. Mater. Sci. Mater. Electron. **34**(26), 1826 (2023). 10.1007/s10854-023-11183-2

