# Supplementary material for: Crystallographic Engineering Enables Fast Low-Temperature Ion Transport of TiNb2O7 for Cold-Region Lithium-Ion Batteries
Source: Nanomicro Lett. 2026 Jan 1;18:91. doi: 10.1007/s40820-025-01949-0 (PMC12756213; doi:10.1007/s40820-025-01949-0)
Supplement: Supplementary file 1 — Supplementary file1 (DOC 29726 KB) [file 40820_2025_1949_MOESM1_ESM.doc]

Supporting Information for

**Crystallographic Engineering Enables Fast Low-Temperature Ion Transport of TiNb_2_O_7_ for Cold-Region Lithium-Ion Batteries**

Lihua Wei ^1#^, Shenglu Geng ^1#^, Hailu Liu ^1^, Liang Deng ^1^, Yiyang Mao ^1^, Yanbin Ning ^1^, Biqiong Wang ^2^*, Yueping Xiong ^1^, Yan Zhang ^1^*, Shuaifeng Lou ^1^*

^1^ State Key Laboratory of Space Power-Sources, School of Chemistry and Chemical Engineering, Harbin Institute of Technology, Harbin 150001, P. R. China

^2^ China Tower Corporation Limited No.9 Dongran North Street, Haidian District Beijing 100089, P. R. China

^#^Lihua Wei and Shenglu Geng contributed equally to this work.

*Corresponding authors. E-mail: [wangbq9@chinatowercom.cn](mailto:wangbq9@chinatowercom.cn) (Biqiong Wang); [zhangyhit@hit.edu.cn](mailto:zhangyhit@hit.edu.cn) (Yan Zhang); [shuaifeng.lou@hit.edu.cn](mailto:shuaifeng.lou@hit.edu.cn) (Shuaifeng Lou)

**Supplementary Figures and Tables**


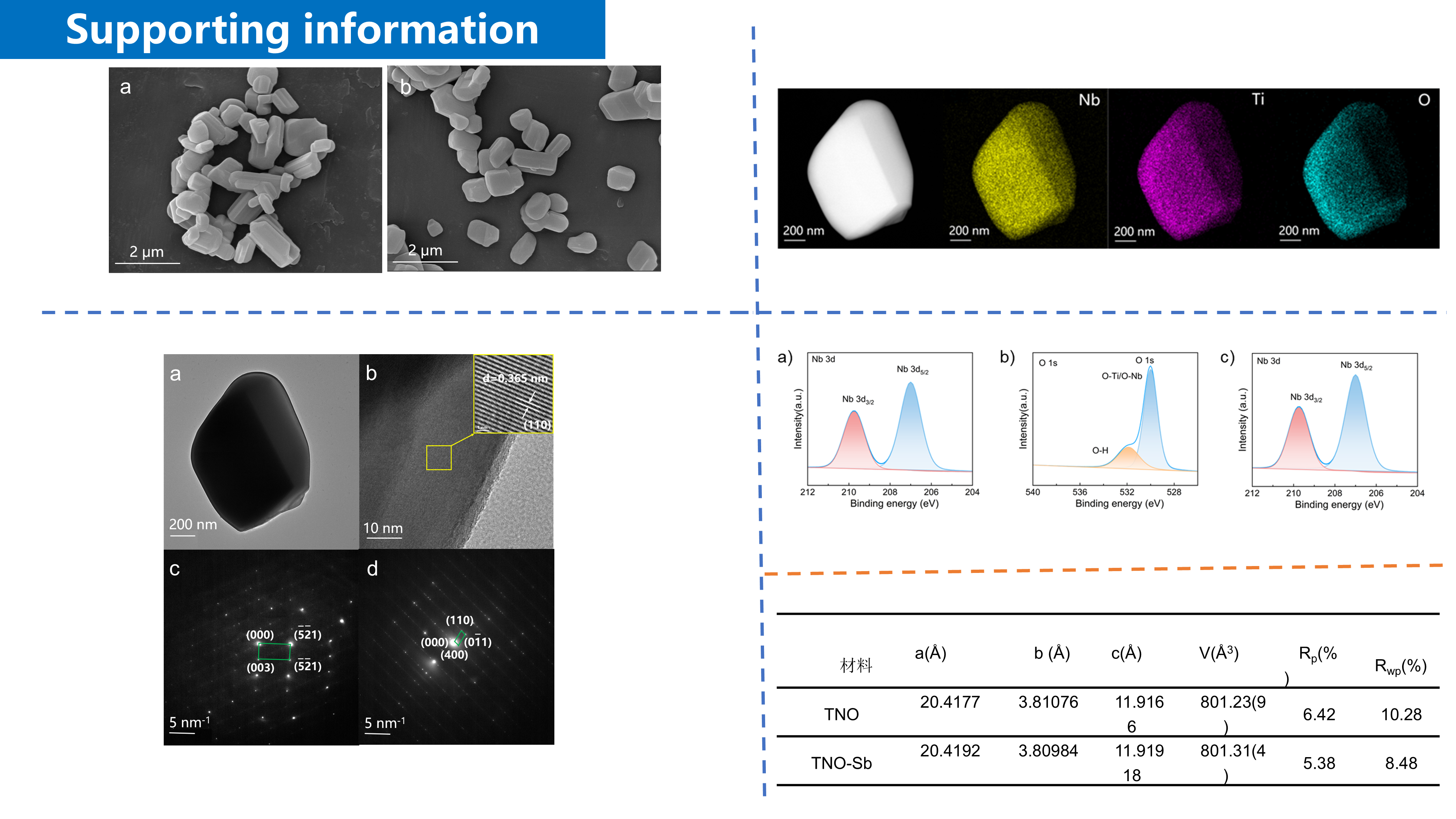


**Fig. S1** SEM images of **a** TNO and **b** TNO-Sb/Nb


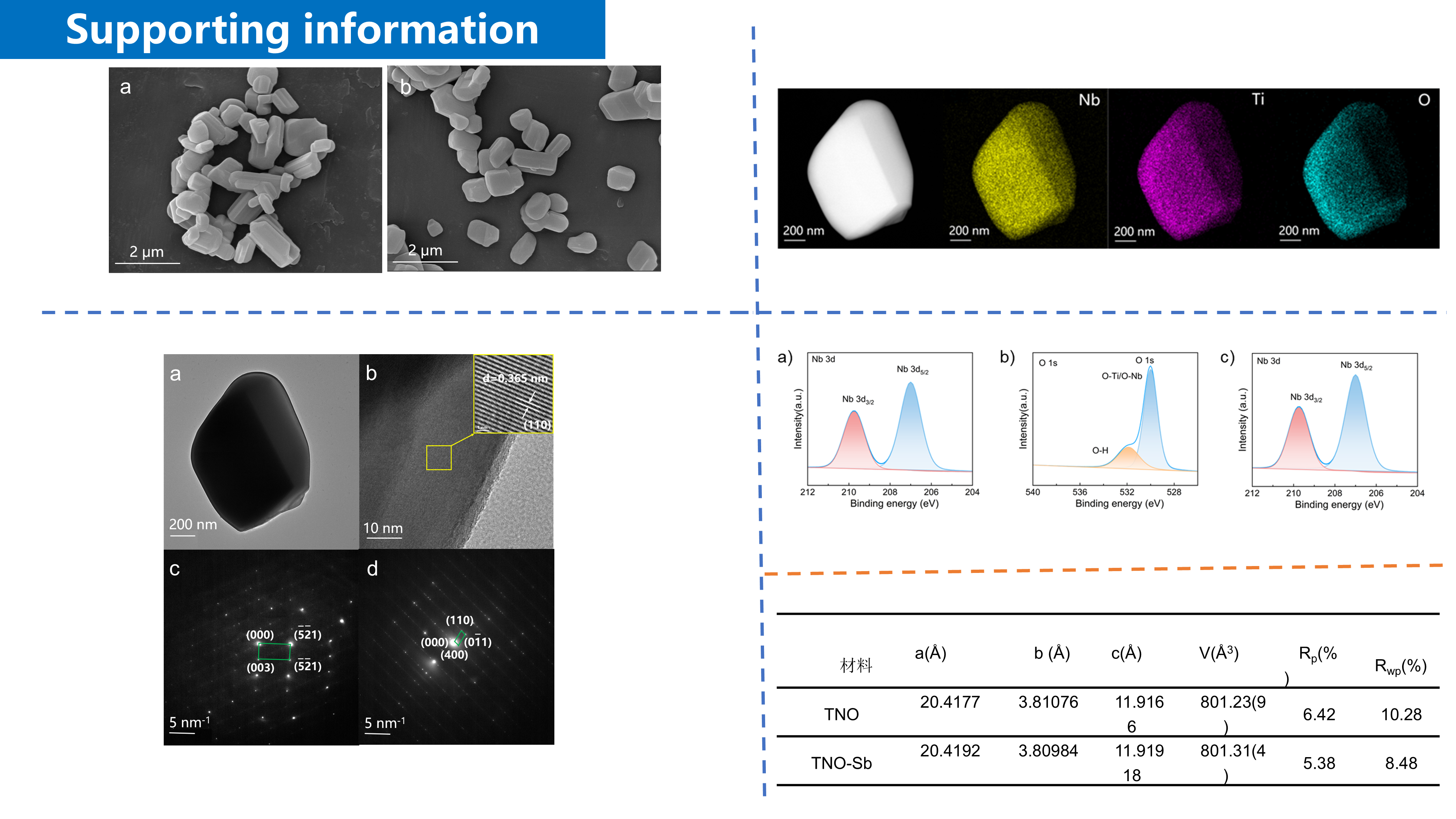


**Fig. S2 a** TEM image of the TNO microrods, **b** HRTEM image of the TNO microrods, the corresponding corresponding SEAD patterns of the **c** TNO and **d** TNO-Sb/Nb

**
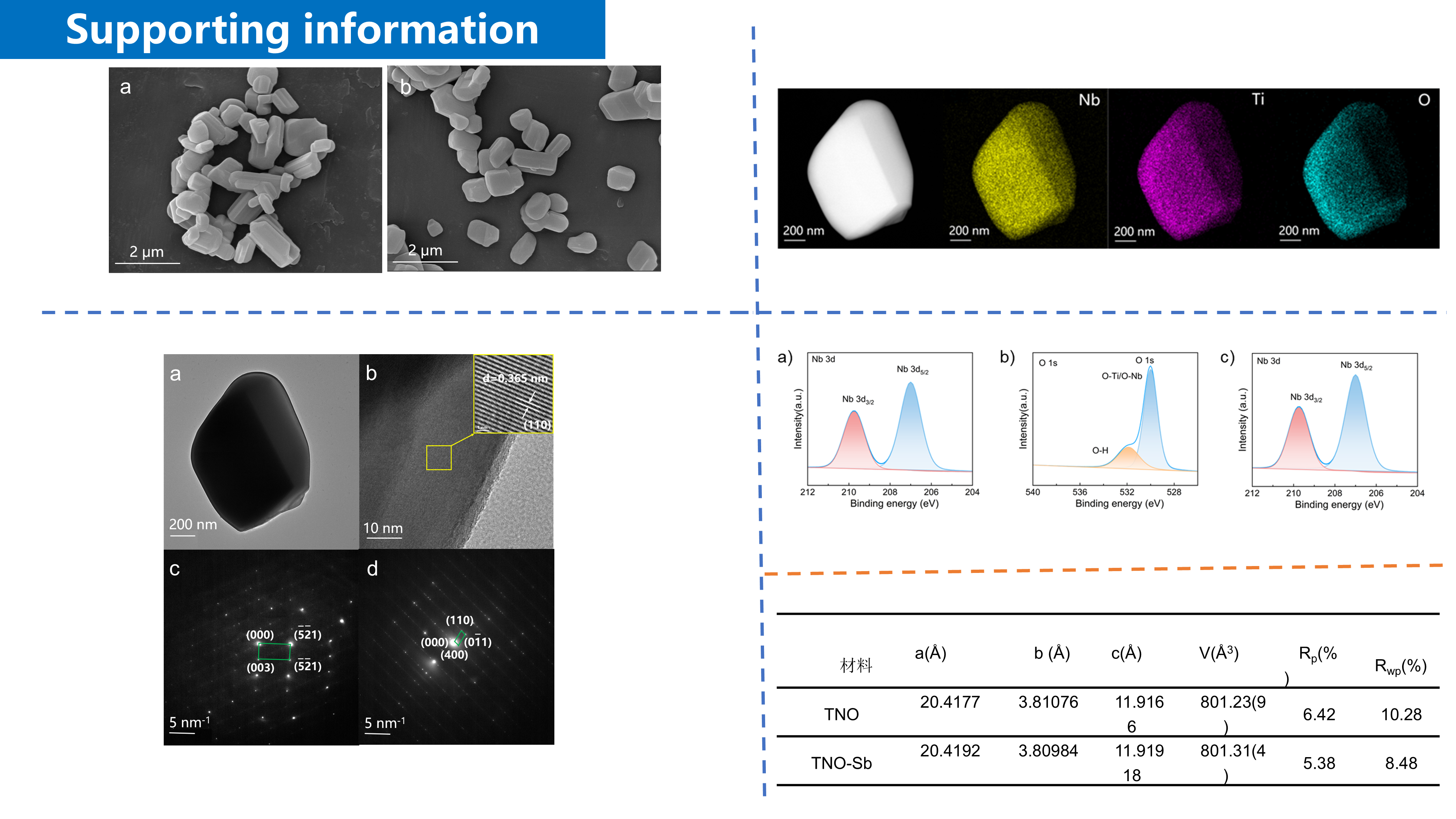
**

**Fig. S3** STEM image and the corresponding EDS mappings of TNO


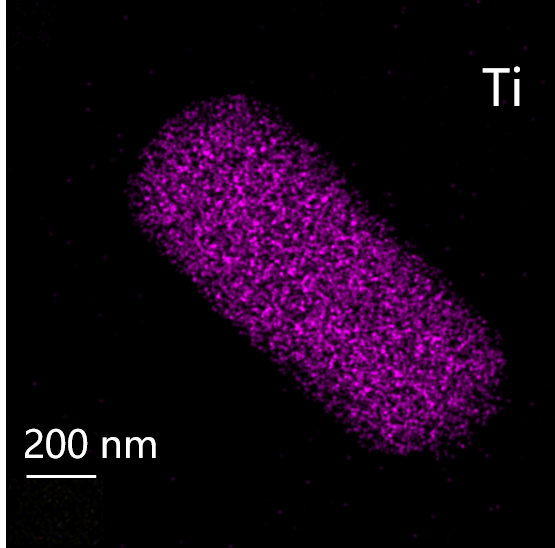


**Fig. S4** EDS mapping of elemental Ti concentrations on the surface of TNO-Sb/Nb


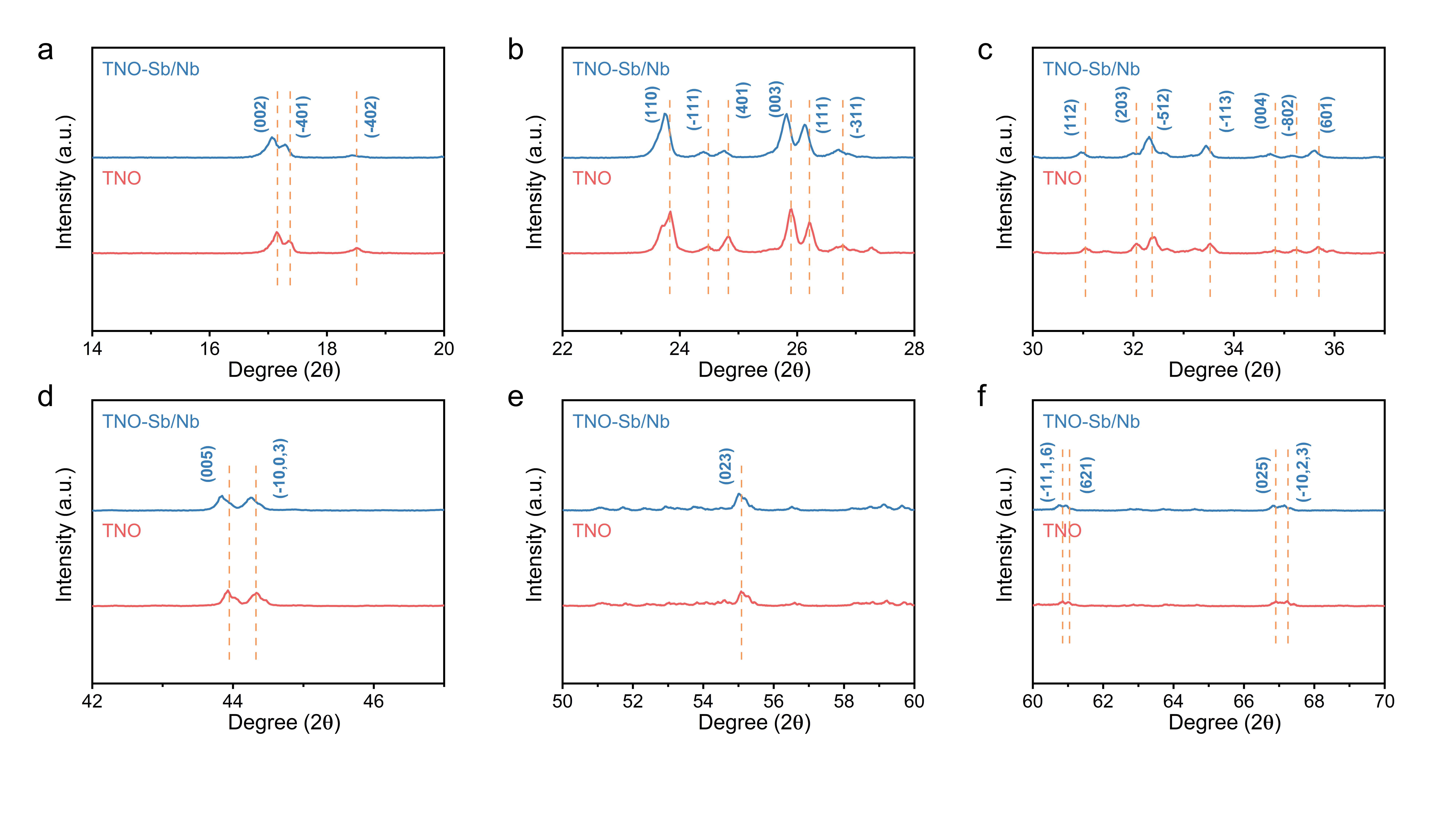


**Fig. S5** The XRD partial enlarged views of TNO and TNO-Sb/Nb


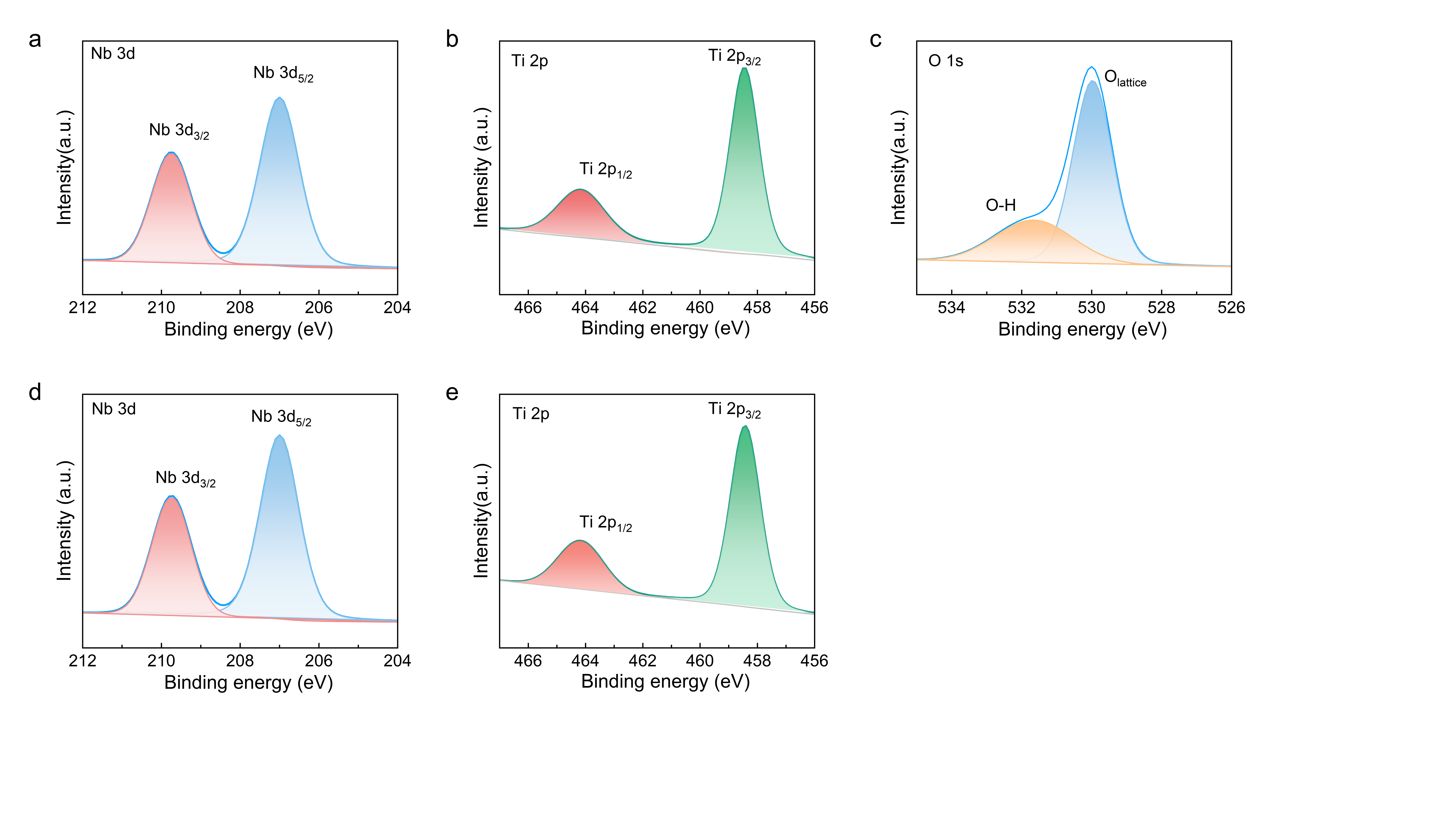


**Fig. S6** XPS high-resolution spectra of **a-c** TNO and **d, e** TNO-Sb/Nb


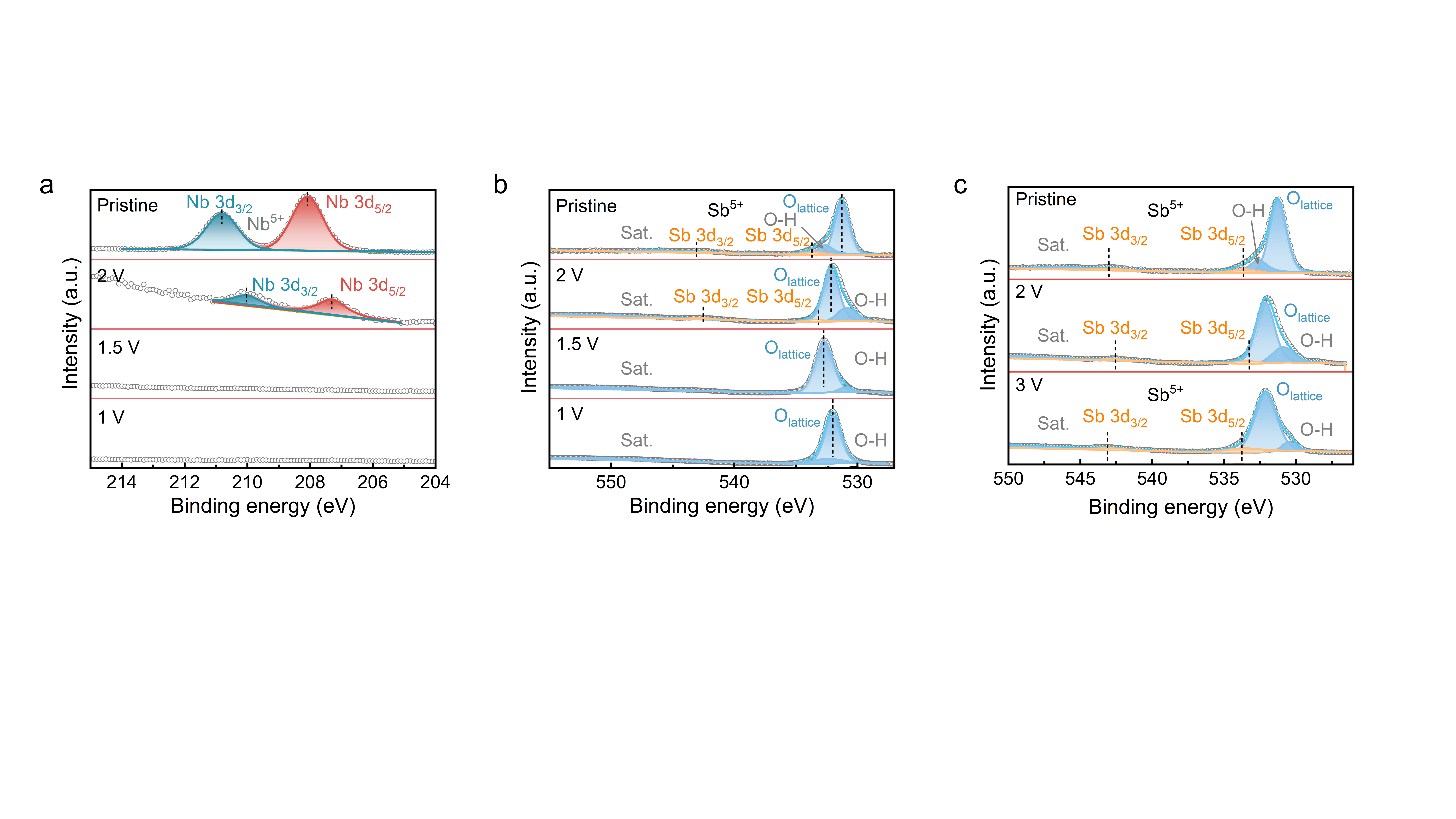


**Fig. S7** XPS high-resolution spectra of as-prepared TNO-Sb/Nb at different states of charge (the first cycle)


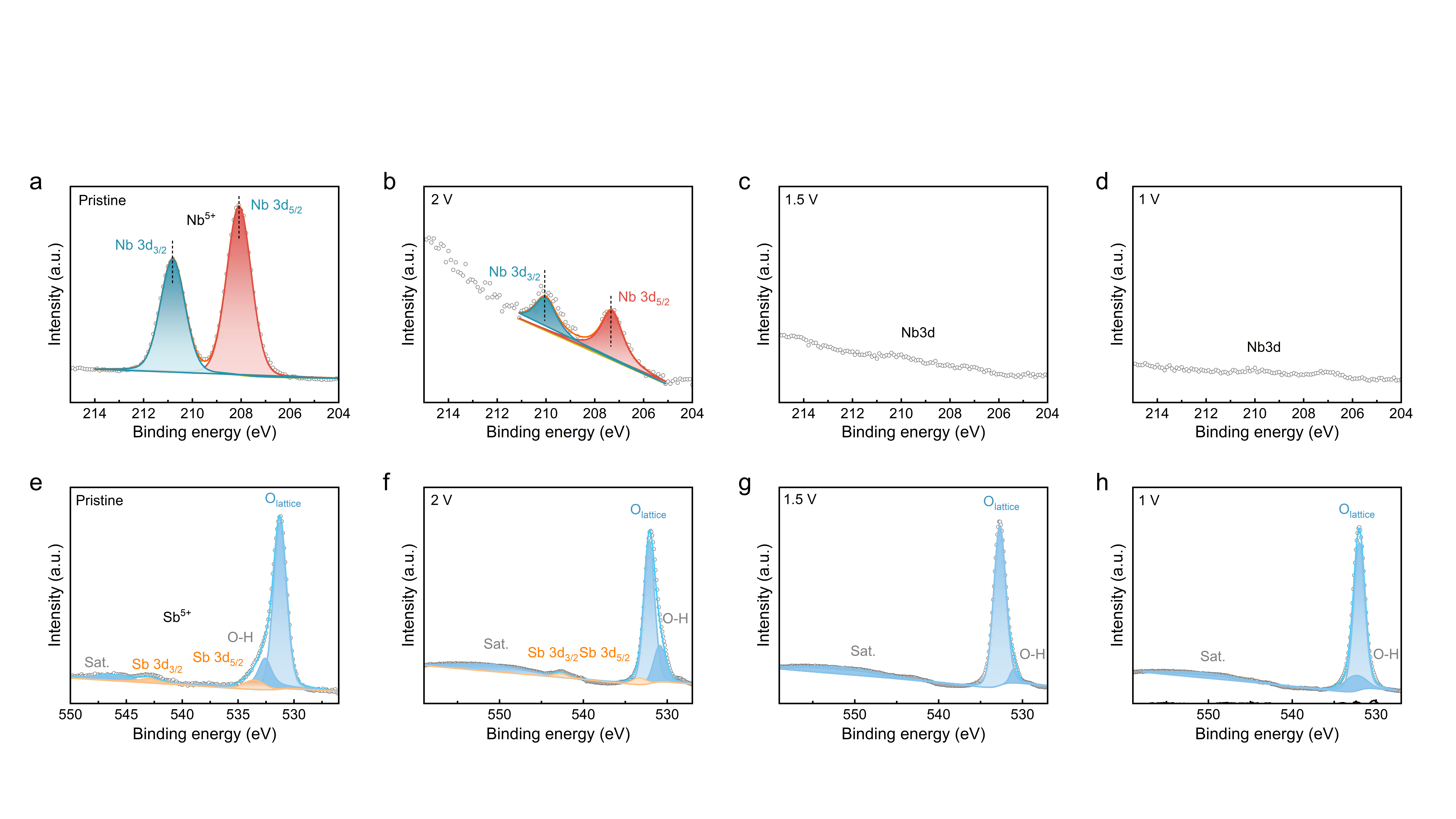


**Fig. S8** The XPS spectra detail of Nb 3d and Sb 3d for TNO-Sb/Nb at different discharge states

**
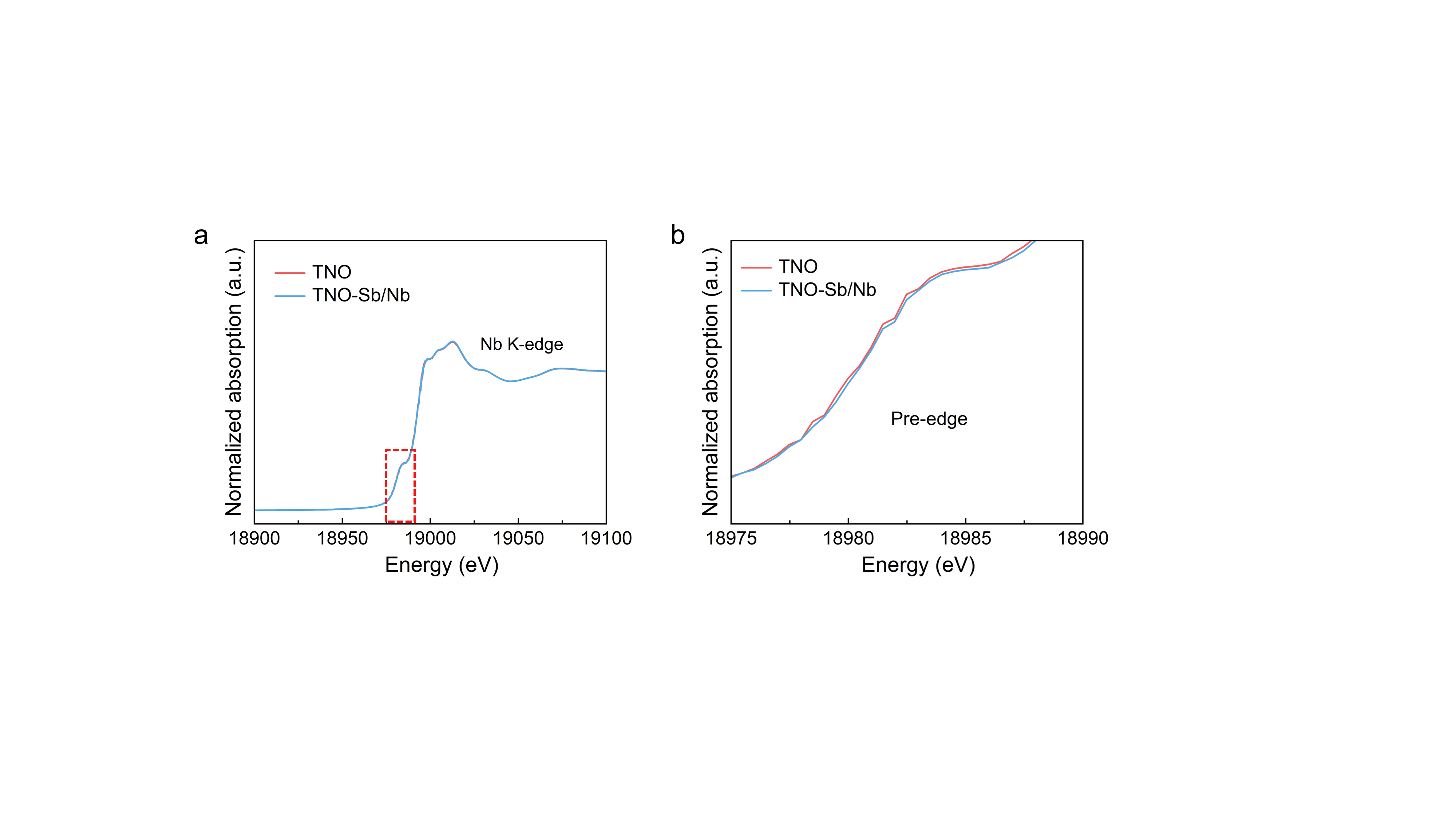
**

**Fig. S9 a** Nb K-edge XAS spectra of the TNO and TNO-Sb/Nb. **b** X-ray absorption near edge spectroscopy (XANES) spectra of the Nb K-edge of the TNO and TNO-Sb/Nb

**
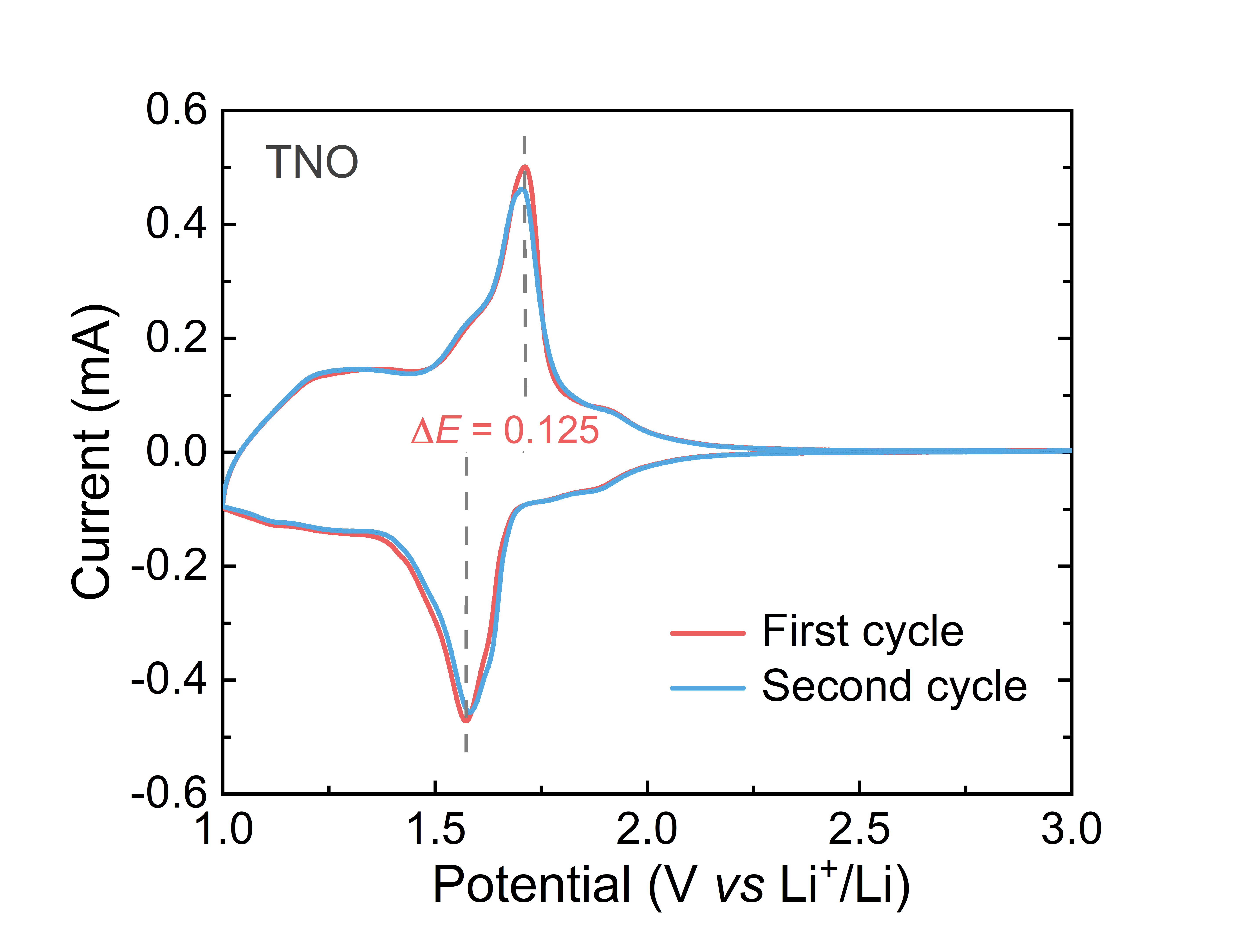
**

**Fig.** **S10** CV curves of TNO within 1-3 V during the first two cycles


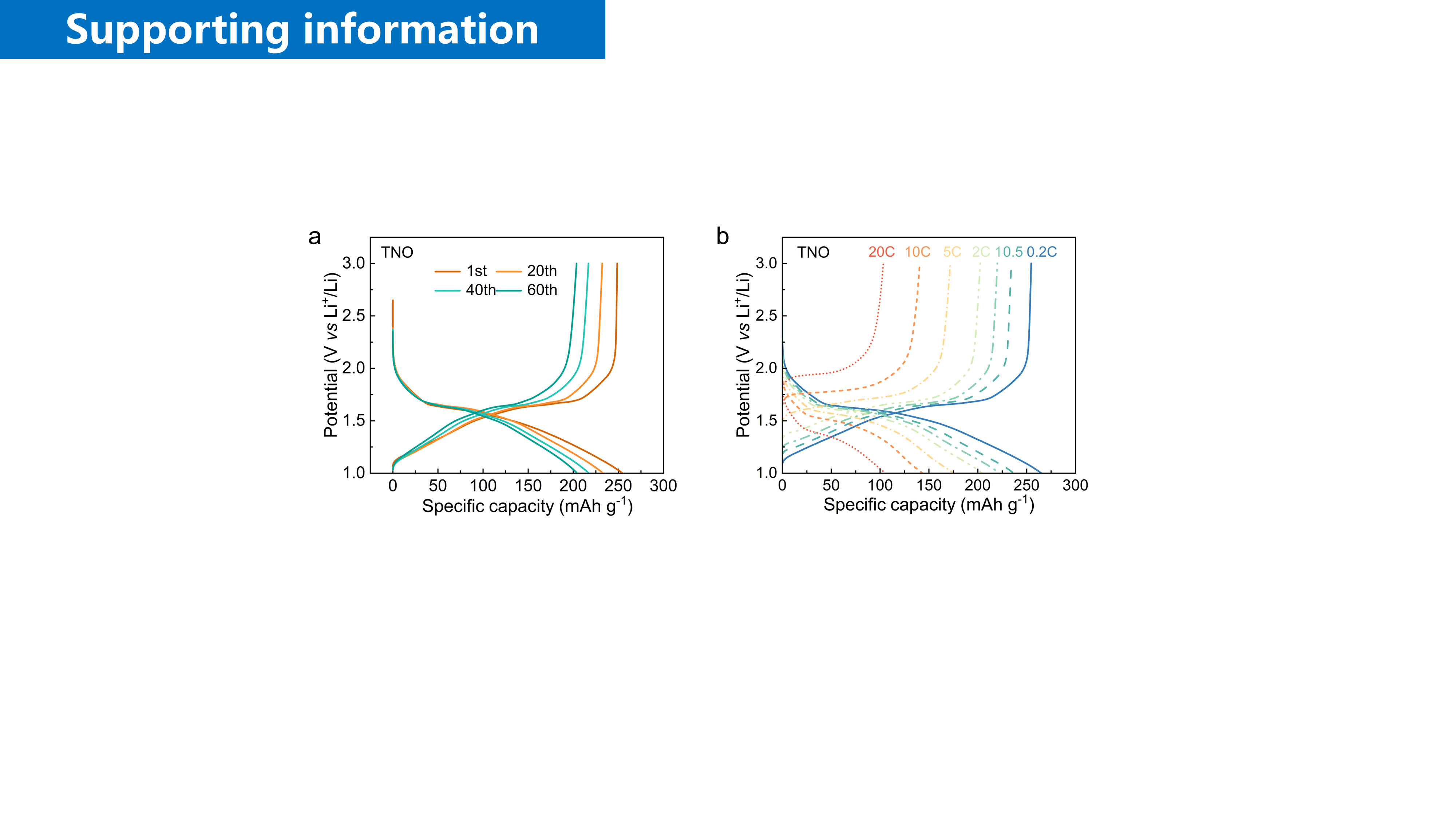


**Fig. S11 a** Charge-discharge curves of TNO cells with different number of cycles at 0.1C at 1-3 V. **b** Charge-discharge curves of TNO at various current densities


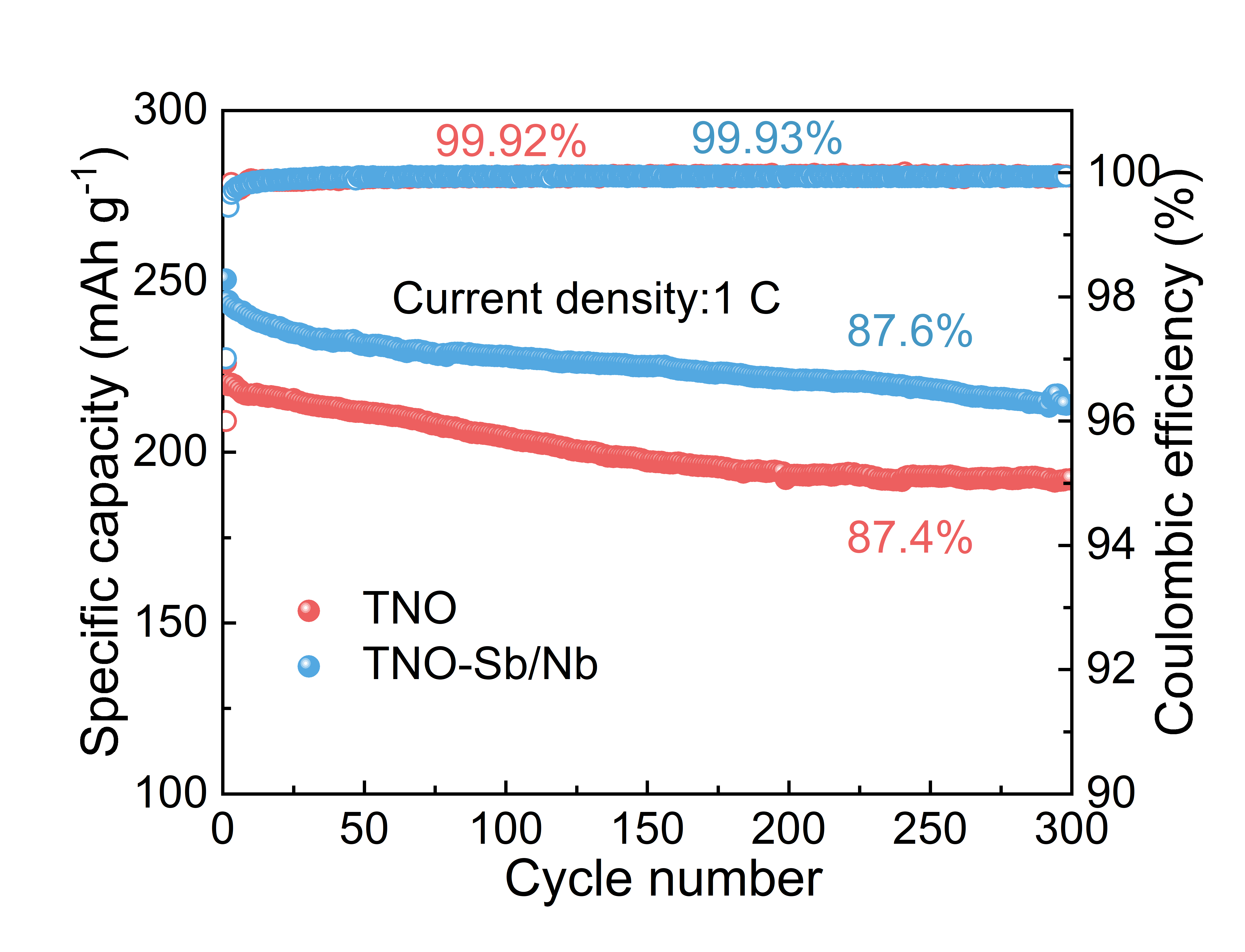


**Fig. S12** Cycling performances of TNO and TNO-Sb/Nb at 1 C


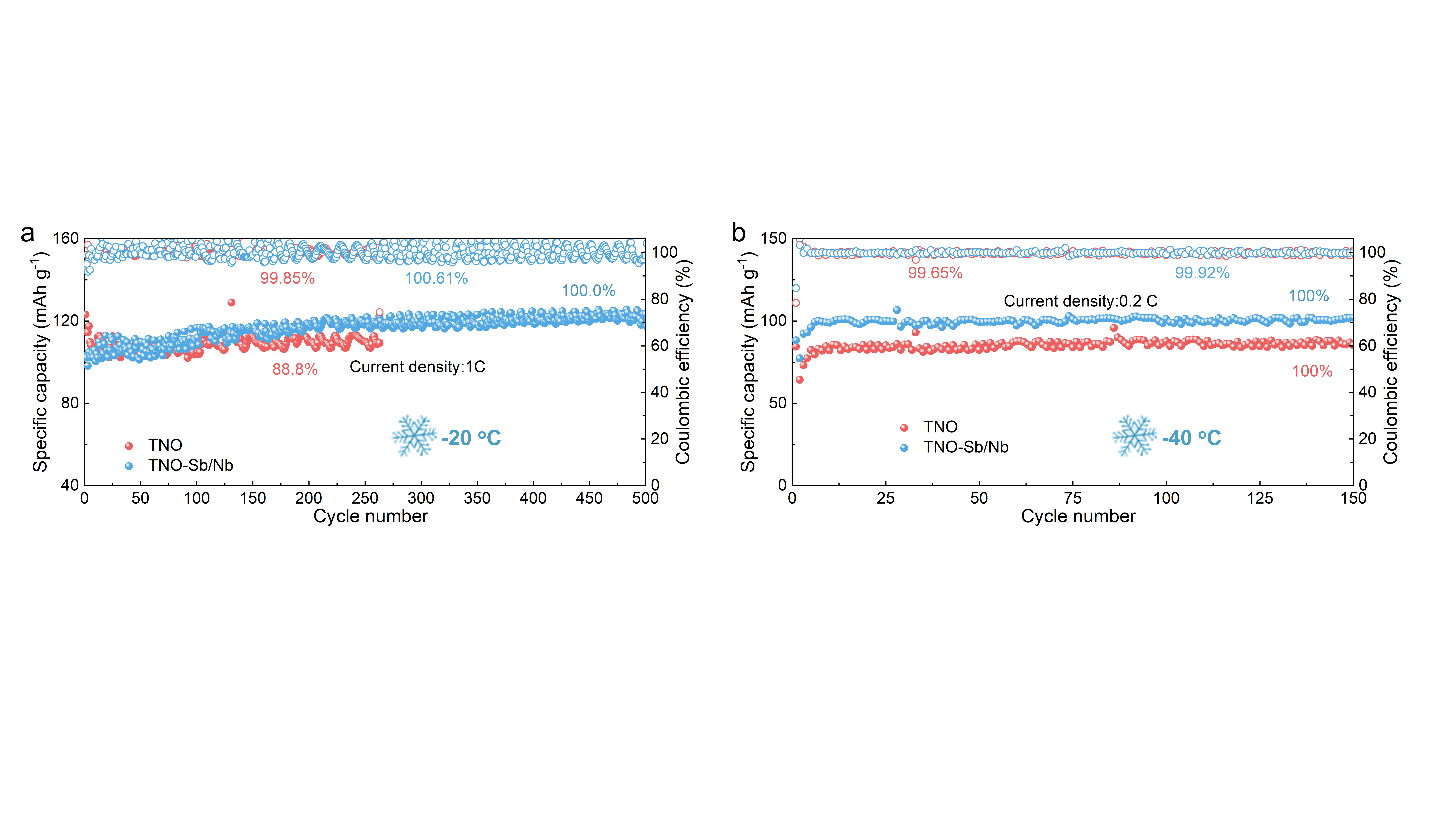


**Fig. S13** Cycling performance of TNO and TNO-Sb/Nb at 1 C in **a** -20 °C and **b** -40 °C


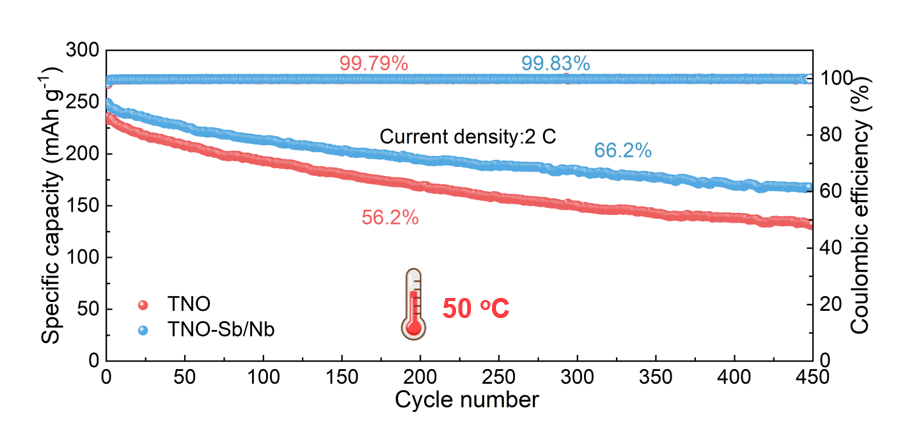


**Fig. S14** High-temperature cycling performance of TNO and TNO-Sb/Nb at 2 C in 50 °C


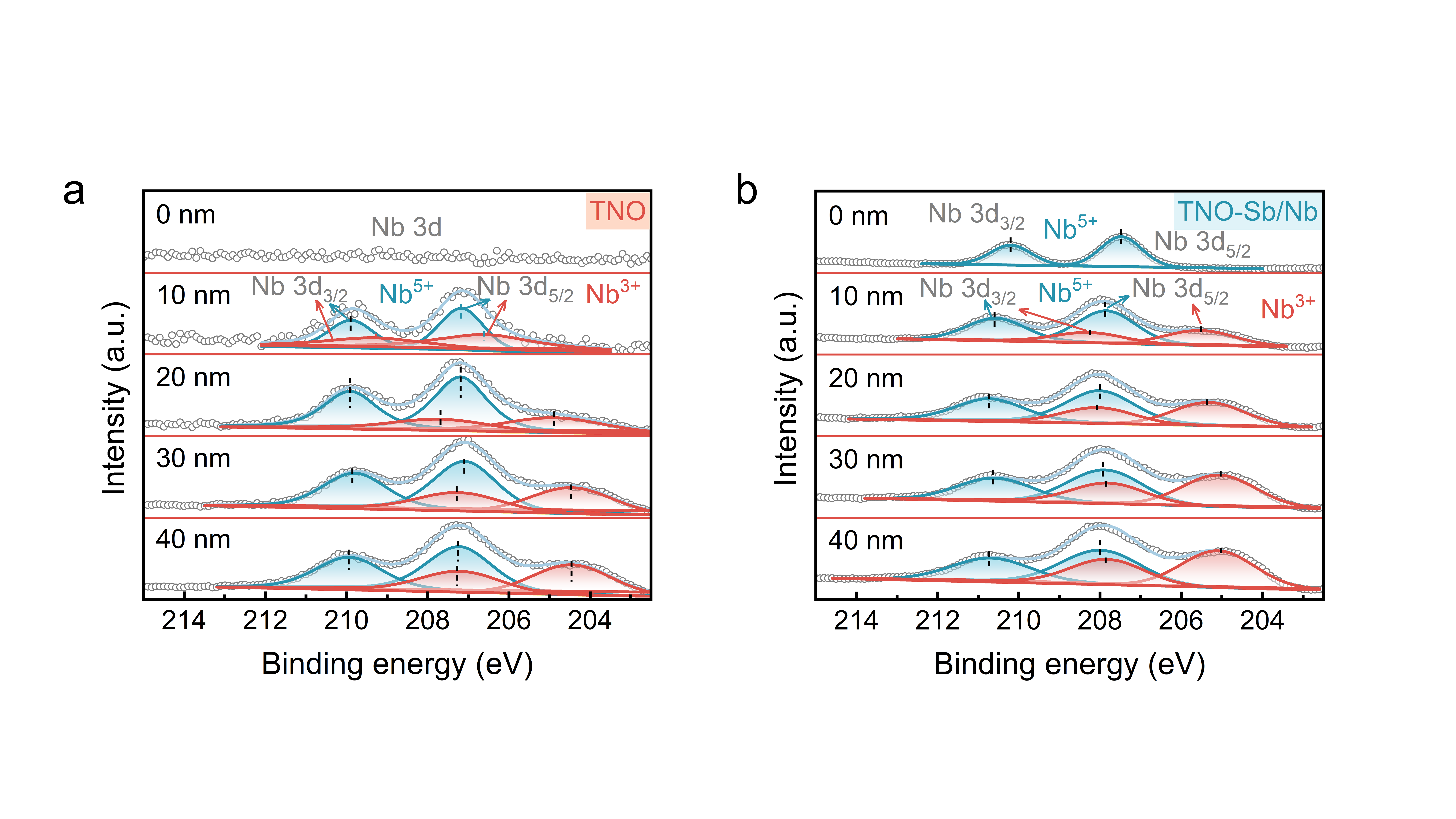


**Fig. S15** XPS etching spectra of Nb 3d for **a** TNO and **b** TNO-Sb/Nb


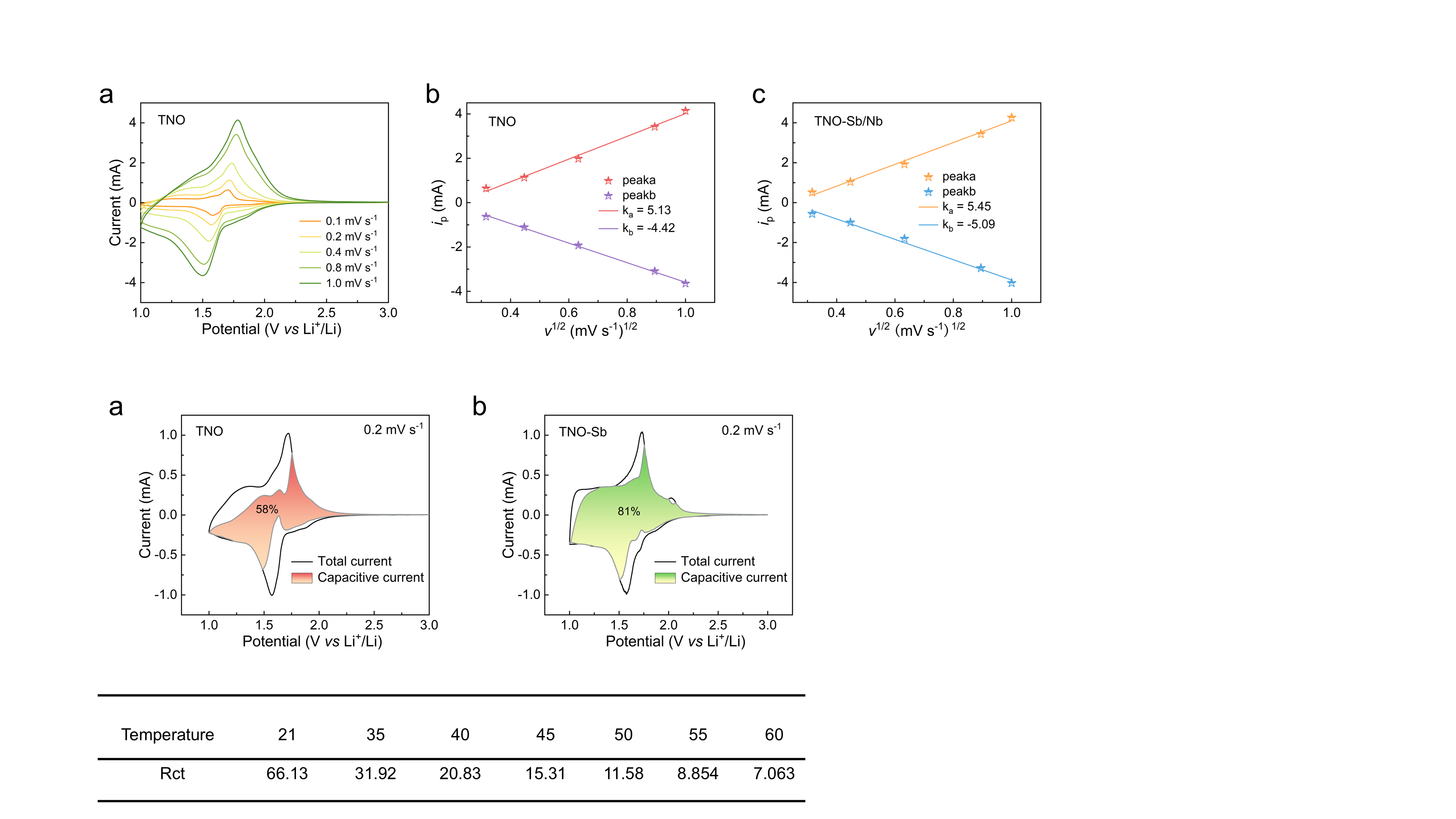


**Fig. S16 a** Cyclic voltammograms of TNO at different scan rates. The linear fitting results of peak current versus square root of the scan rate at the largest oxidation and reduction peaks of **b** TNO and **c** TNO-Sb/Nb electrodes


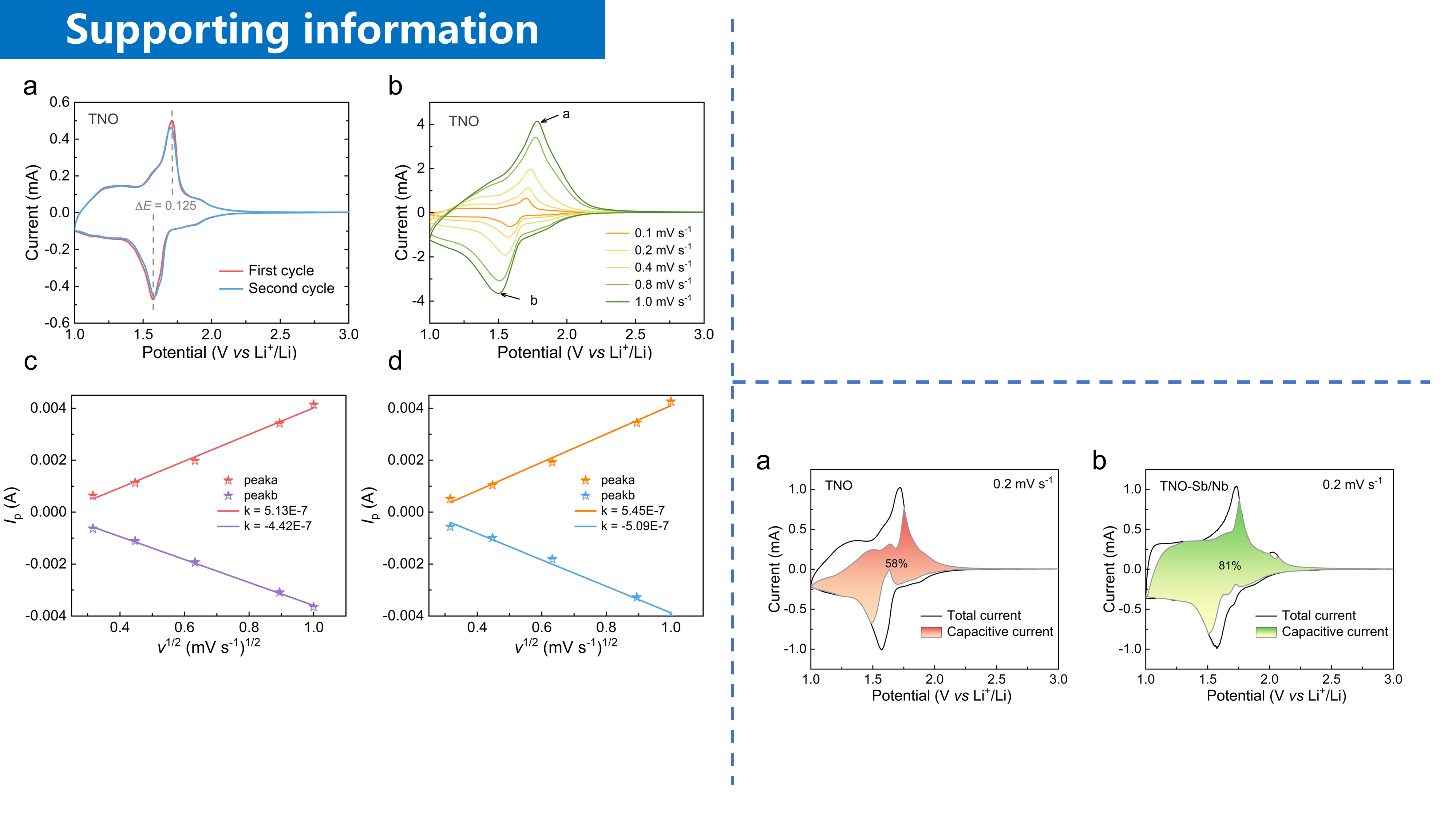


**Fig. S17 a** Pseudocapacitive contributions of TNO at 0.2 mV s^-1^. **b** Pseudocapacitive contributions of TNO-Sb/Nb at 0.2 mV s^-1^


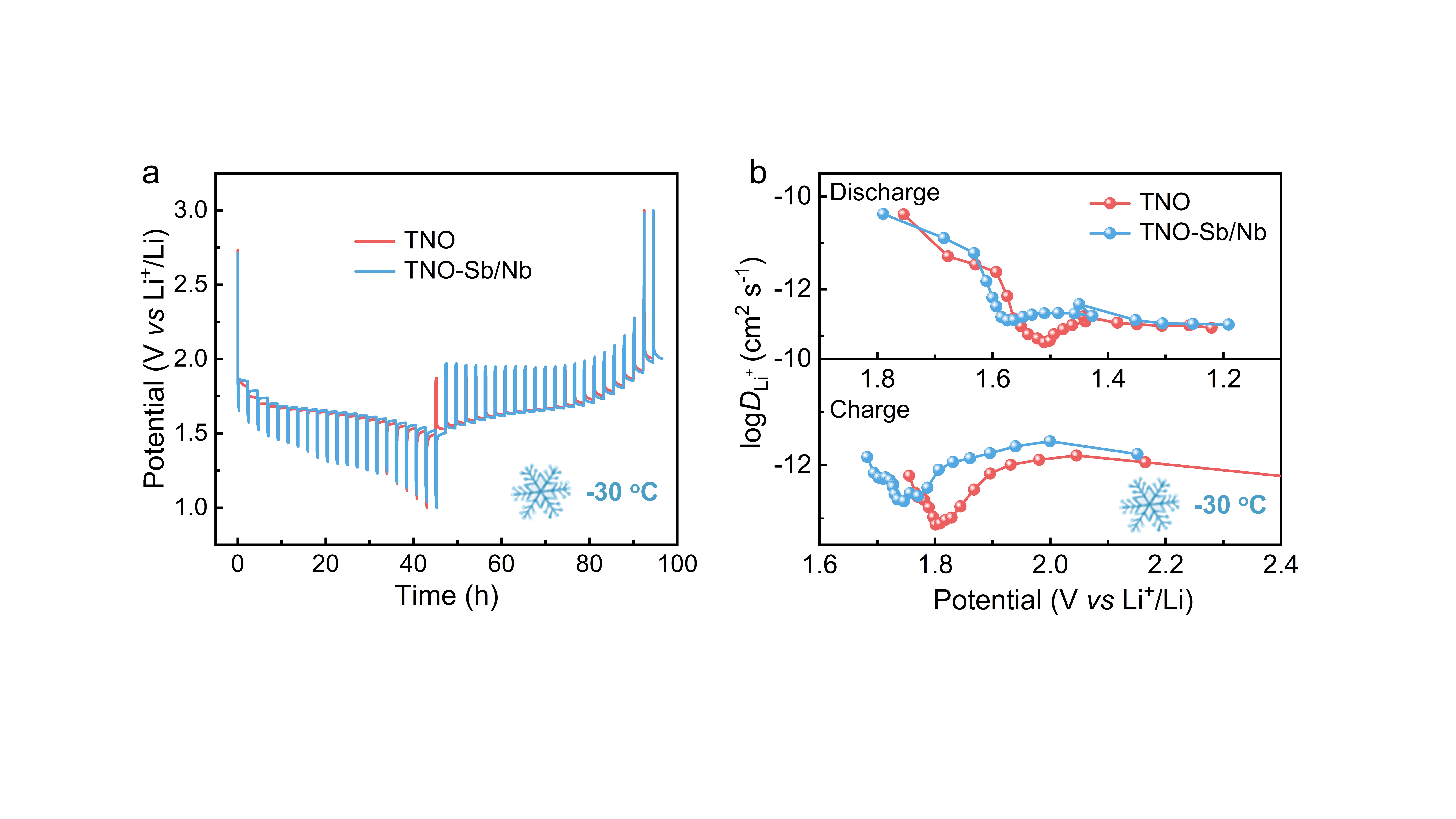


**Fig. S18** **a** GITT curves and **b** Li^+^ diffusion coefficients of TNO and TNO-Sb/Nb at -30 °C

**
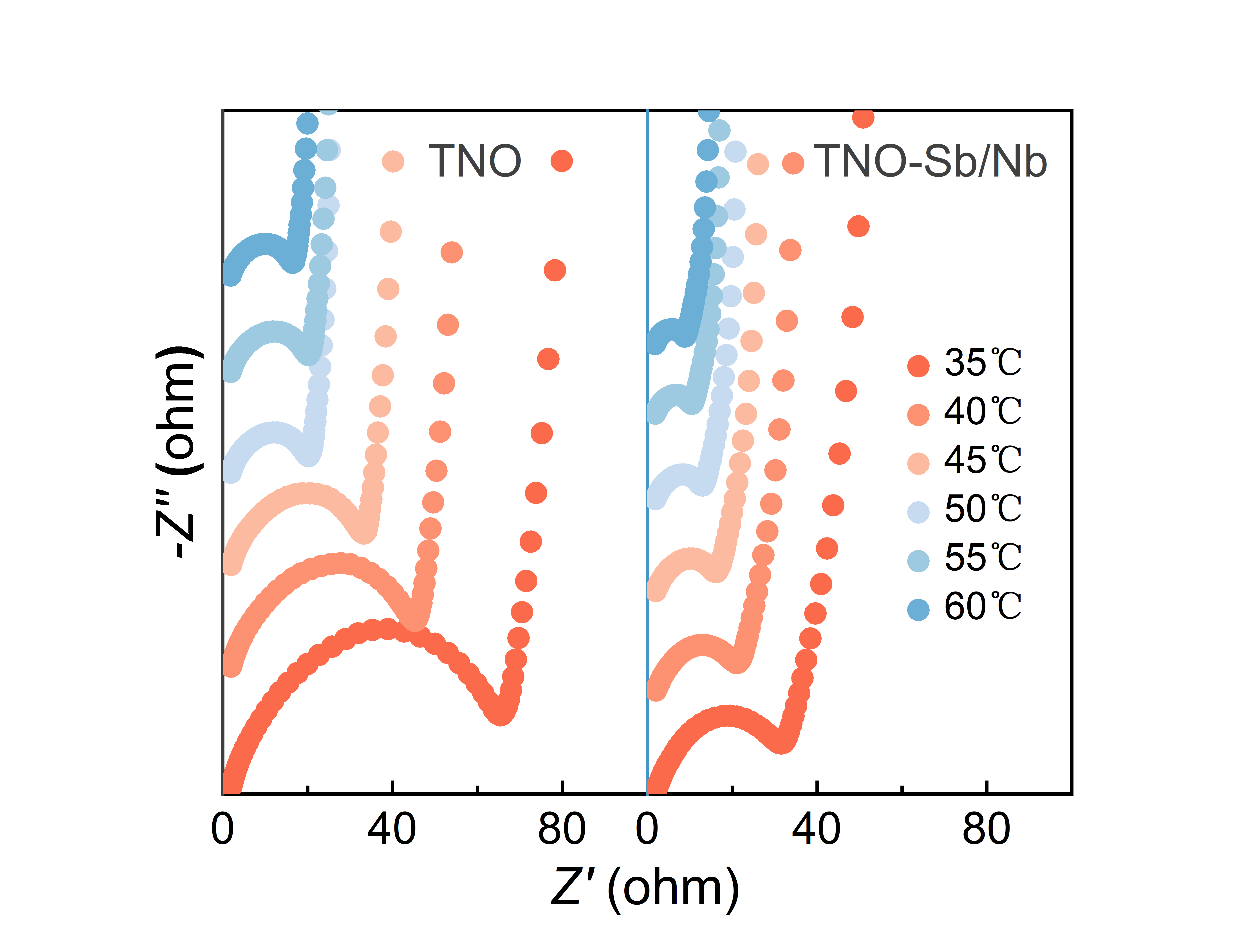
**

**Fig.** **S19** EIS results of TNO and TNO-Sb/Nb at various temperatures


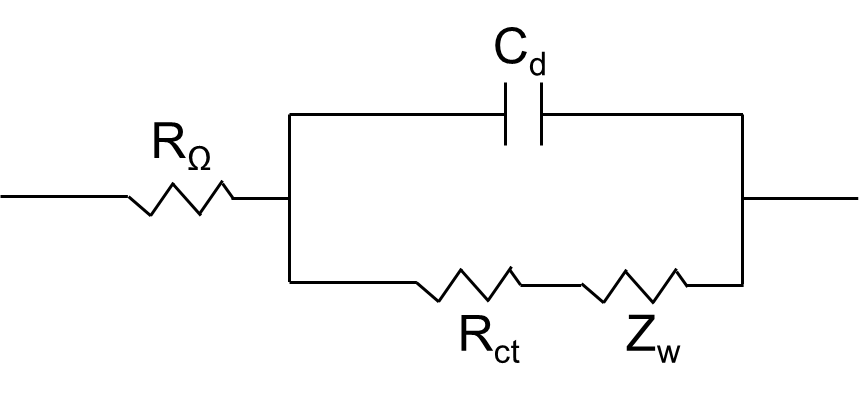


**Fig.** **S20** The equivalent circuit according to the EIS patterns


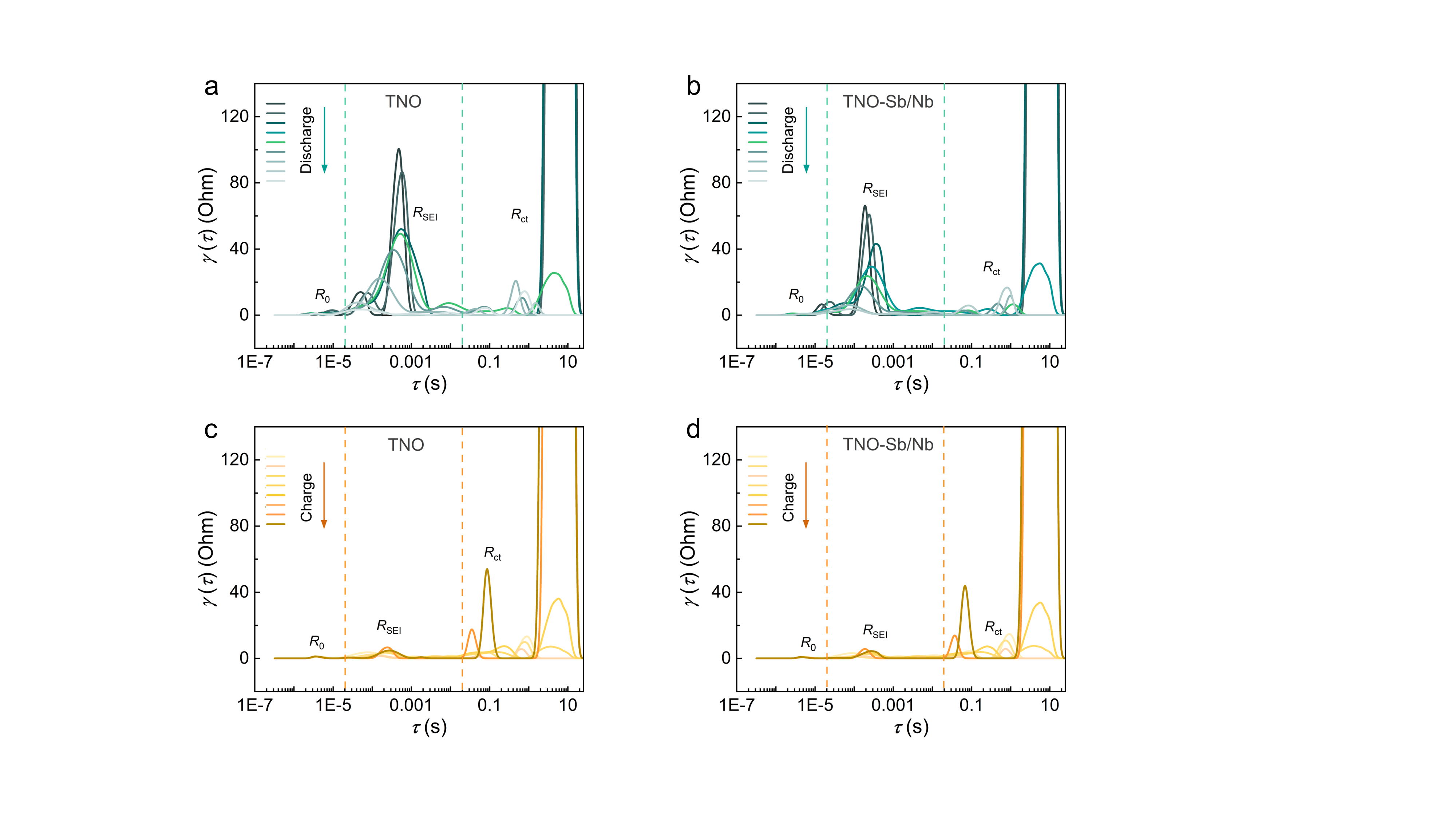


**Fig.** **S21** DRT profiles of **a**, **c** TNO and **b**, **d** TNO-Sb/Nb measured at different SOC


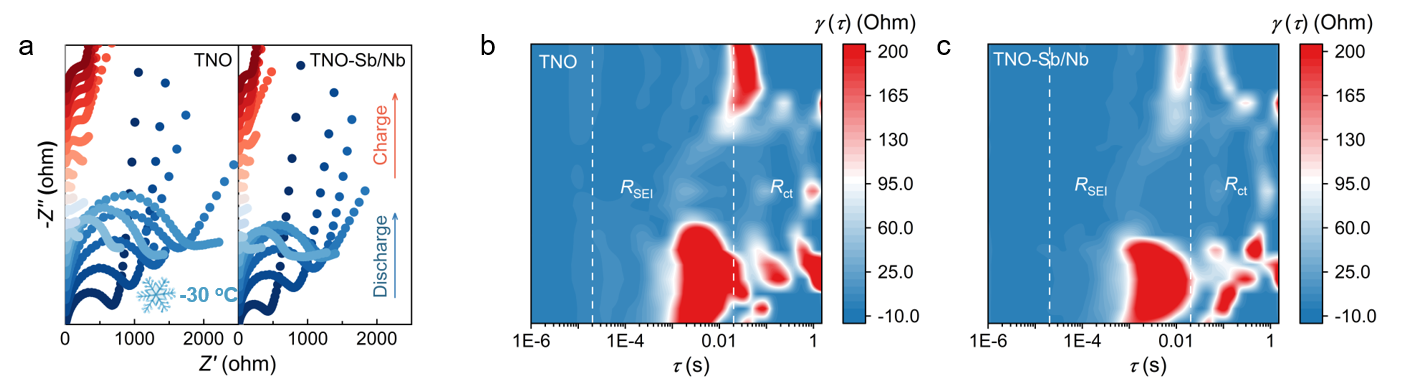


**Fig. S22 a** The low-temperature EIS evolution at different potentials. The corresponding two-dimensional DRT diagram for **b** TNO and **c** TNO-Sb/Nb


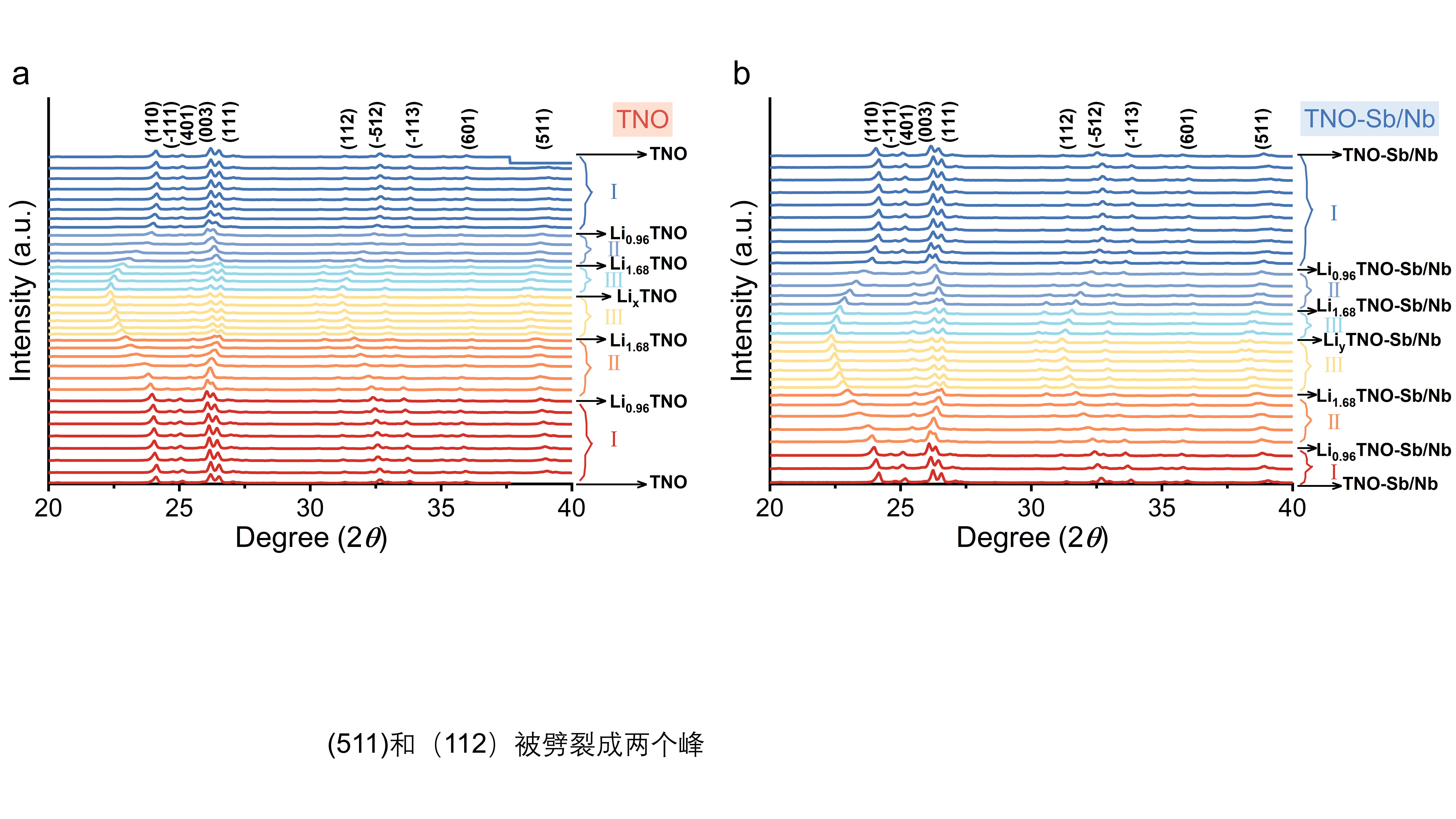


**Fig. S23** In-situ XRD patterns of **a** TNO and **b** TNO-Sb/Nb


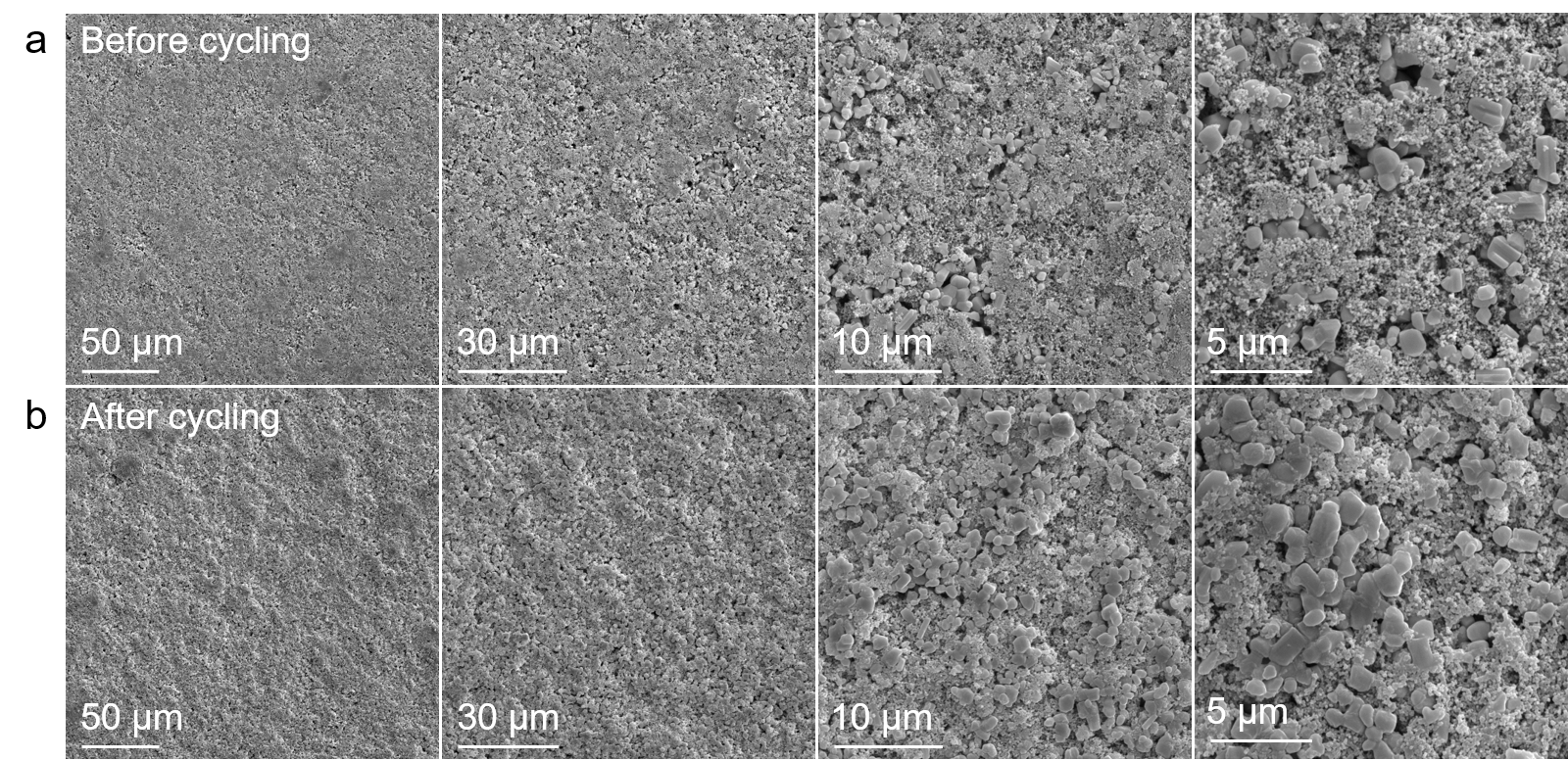


**Fig. S24** The SEM images of TNO-Sb/Nb electrode **a** before and **b** after 500 cycles at -30 ^o^C


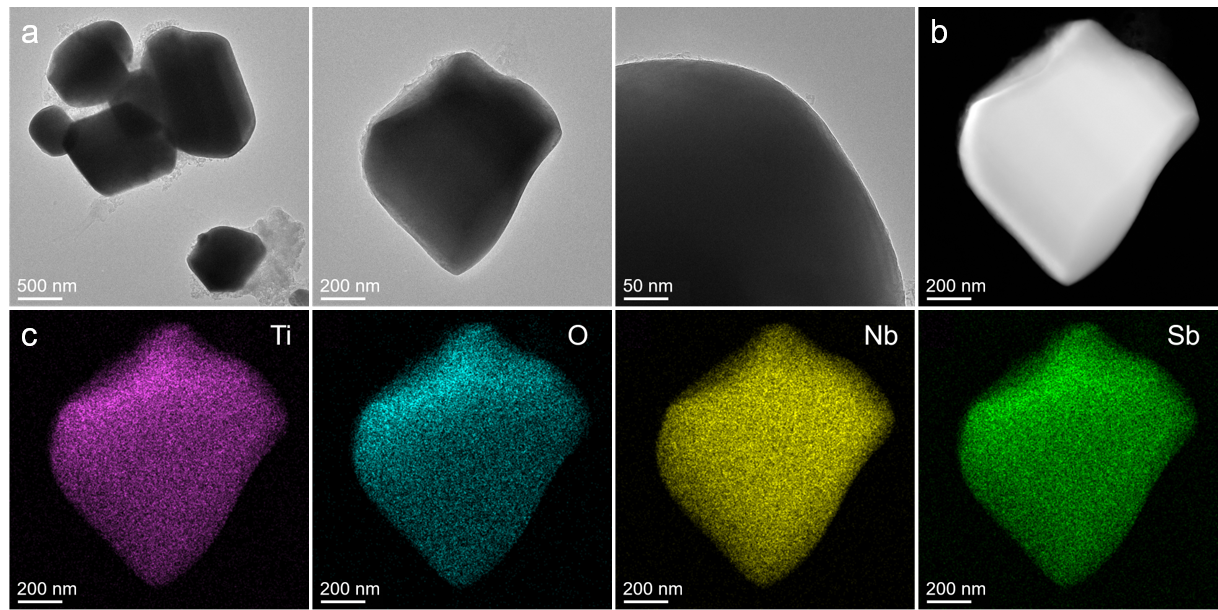


**Fig. S25** **a** The TEM images, **b** the STEM image, and **c** the elemental mapping of the TNO-Sb/Nb microrods after 500 cycles at -30 ^o^C


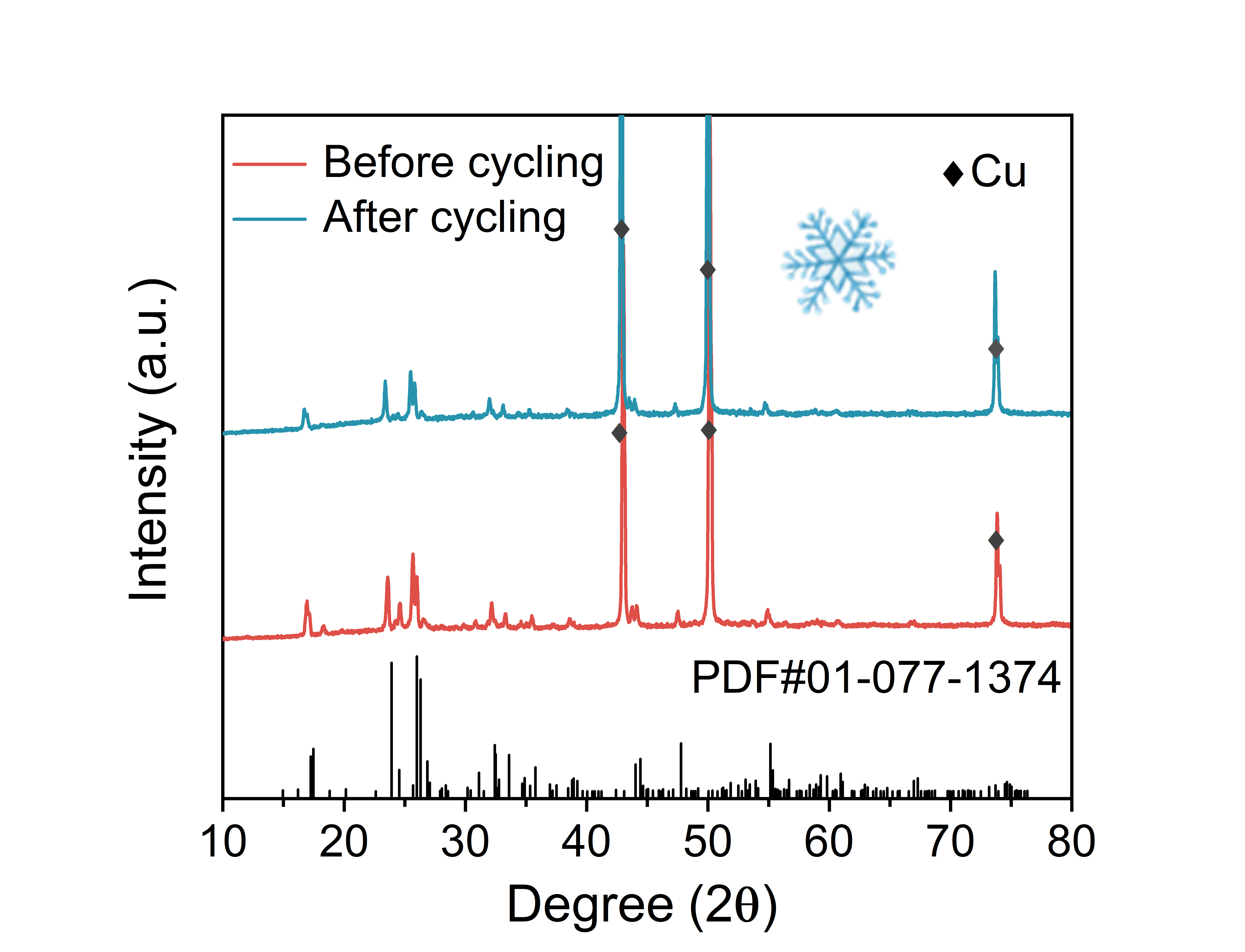


**Fig. S26** The XRD patterns before and after 500 cycles at -30 ^o^C

**Table S1** ICP results of TNO and TNO-Sb/Nb

| **material** | **Ti (mol)** | **Nb (mol)** | **Sb (mol)** |
| --- | --- | --- | --- |
| TNO | 1 | 2.2634 | 0 |
| TNO-Sb/Nb | 1 | 2.2888 | 0.0096 |

**Table** **S2** Rietveld refinement results of TNO and TNO-Sb/Nb

| **Material** | ***a* (Å)** | ***b* (Å)** | ***c* (Å)** | ***V* (Å^3^)** | **R_p_ (%)** | **R_wp_ (%)** |
| --- | --- | --- | --- | --- | --- | --- |
| TNO | 20.4177 | 3.8107 | 11.9166 | 801.23(9) | 6.42 | 10.28 |
| TNO-Sb/Nb | 20.4192 | 3.8098 | 11.9192 | 801.31(4) | 5.38 | 8.48 |

**Table** **S3** Equivalent circuit fitting data of Nyquist spectra

| **Temperature (^o^C)** | **21** | **30** | **35** | **40** | **45** | **50** | **55** | **60** |
| --- | --- | --- | --- | --- | --- | --- | --- | --- |
| *R*_ct_ of TNO | 165.8 | 95.8 | 65.7 | 44.4 | 33.7 | 19.0 | 19.0 | 15.5 |
| *R*_ct_ of TNO-Sb/Nb | 66.1 | 39.4 | 31.9 | 20.8 | 15.3 | 11.6 | 8.9 | 7.1 |

**Table S4** Results of nanoindentation test on TNO

| **point** | **1** | **2** | **3** | **4** | **Mean** |
| --- | --- | --- | --- | --- | --- |
| Avg Modulus | 4.3 | 1.3 | 11.4 | 4.6 | 5.4 |
| Avg Hardness | 0.05 | 0.02 | 0.16 | 0.03 | 0.07 |

**Table S5** Results of nanoindentation test on TNO-Sb/Nb

| **point** | **1** | **2** | **3** | **4** | **Mean** |
| --- | --- | --- | --- | --- | --- |
| Avg Modulus | 1.5 | 13.7 | 12.2 | 10.6 | 9.5 |
| Avg Hardness | 0.01 | 0.18 | 0.2 | 0.14 | 0.13 |
